# Stable Expression of *mtlD* Gene Imparts Multiple Stress Tolerance in Finger Millet

**DOI:** 10.1371/journal.pone.0099110

**Published:** 2014-06-12

**Authors:** Ramanna Hema, Ramu S. Vemanna, Shivakumar Sreeramulu, Chandrasekhara P. Reddy, Muthappa Senthil-Kumar, Makarla Udayakumar

**Affiliations:** 1 Department of Crop Physiology, University of Agricultural Sciences, GKVK, Bangalore, India; 2 National Institute of Plant Genome Research (NIPGR), Aruna Asaf Ali Marg, New Delhi, India; Kansas State University, United States of America

## Abstract

Finger millet is susceptible to abiotic stresses, especially drought and salinity stress, in the field during seed germination and early stages of seedling development. Therefore developing stress tolerant finger millet plants combating drought, salinity and associated oxidative stress in these two growth stages is important. Cellular protection through osmotic adjustment and efficient free radical scavenging ability during abiotic stress are important components of stress tolerance mechanisms in plants. Mannitol, an osmolyte, is known to scavenge hydroxyl radicals generated during various abiotic stresses and thereby minimize stress damage in several plant species. In this study transgenic finger millet plants expressing the mannitol biosynthetic pathway gene from bacteria, mannitol-1-phosphate dehydrogenase (*mtlD*), were developed through *Agrobacterium tumefaciens*-mediated genetic transformation. *mtlD* gene integration in the putative transgenic plants was confirmed by Southern blot. Further, performance of transgenic finger millet under drought, salinity and oxidative stress was studied at plant level in T_1_ generation and in T_1_ and T_2_ generation seedlings. Results from these experiments showed that transgenic finger millet had better growth under drought and salinity stress compared to wild-type. At plant level, transgenic plants showed better osmotic adjustment and chlorophyll retention under drought stress compared to the wild-type. However, the overall increase in stress tolerance of transgenics for the three stresses, especially for oxidative stress, was only marginal compared to other *mtlD* gene expressing plant species reported in the literature. Moreover, the *Agrobacterium*-mediated genetic transformation protocol developed for finger millet in this study can be used to introduce diverse traits of agronomic importance in finger millet.

## Introduction

Finger millet (*Eleusine coracana*) constitutes ∼12% of the global millet area and is cultivated in more than 25 countries in Africa and Asia. It is an important food crop in India, Nepal and several African countries [1]–[3]. It is an under-utilized but an important cereal crop for livelihood in rural areas in many of these countries [4]. It has nutritional qualities better than that of wheat and other prominent cereal crops [5]–[7]. Over the years several approaches, including utilization of somaclonal variations [8] and conventional plant breeding strategies, were used to develop high-yielding finger millet varieties like GPU28 and Indaf 9 [9]–[11]. However, since the major area of cultivation of these varieties is rain-fed, the potential yields are affected by severe drought stress [12]–[15] and drought-induced oxidative stress [16]. In addition, salinity stress also reduces potential yields [17], [18]. More specifically, seed germination and seedling establishment stages are highly susceptible to drought and salinity stress [12], [18]. Various agronomic practices during sowing and plant establishment in the field [19]–[21] have not improved the productivity of finger millet [12]. Hence, developing plants having higher intrinsic stress tolerance is necessary for yield improvement.

Osmolytes have been attributed to enhance abiotic stress tolerance in plants [22], [23]. Relevance of diverse range of osmolyte compounds including carbohydrate-, sugar alcohol- and amino acid-derived compounds under abiotic stress have been shown [22]–[25]. Developing finger millet plants that can accumulate novel osmolytes is useful for improving stress tolerance. From this perspective, utilizing the existing genetic variability among finger millet germplasm for increased osmolyte production and genetic engineering for a particular osmolyte are approaches that have been attempted in finger millet [16], [26]–[29]. However, incorporating traits from a stress tolerant germplasm line into cultivated variety by conventional breeding is time consuming [30]. Hence, developing transgenic plants expressing novel stress responsive genes has relevance in improving crop performance under abiotic stresses like drought and salinity. To date three research groups have demonstrated genetic transformation in finger millet [17], [31]–[35], however, genetic engineering in this crop has not been routine and not widely employed as a method for crop improvement. One of the reasons for this is lack of efficient and easy genetic transformation protocol. In this direction one focus of this study was to optimize *Agrobacterium*-mediated transformation and plant regeneration from callus in finger millet.

Mannitol, a sugar alcohol, is an important osmolyte and scavenger of reactive oxygen species (ROS). This compound has been reported to impart tolerance to abiotic stresses in different plant species [36]–[39]. Also, apart from protection against free radicals, transgenic Arabidopsis plants producing mannitol have been shown to express various abiotic stress tolerance related genes [40] leading to enhanced protection during several abiotic stresses. A mannitol biosynthetic gene, mannitol-1-phosphate dehydrogenase (*mtlD*), from *Escherichia coli* is known to catalyze mannitol production in various transgenic plants engineered with this gene and also these plants showed enhanced abiotic stress tolerance [41]–[47]. Stress tolerance of these transgenic plants has been shown to be due to either by enhanced osmoprotection or through protection against free radicals that otherwise cause oxidative stress. For example, the transgenic tobacco plants expressing *mtlD* gene targeted to chloroplast showed increased hydroxyl radical scavenging capacity and retention of chlorophyll in leaf tissue under methyl viologen-induced oxidative stress [36].

Finger millet is known to accumulate proline under stress [48], [49], but, mannitol production has not been reported in this plant. However, like tobacco [41], [50], [51], potato [46], petunia [43], eggplant [52], sorghum [53], rice [54] and other plants, the substrate for mtlD enzyme action and pathway for production of mannitol is expected to present in finger millet. This study demonstrates a protocol for *Agrobacterium tumefaciens* (here after referred as *Agrobacterium*)-mediated transformation in finger millet. Also, by using this protocol *mtlD* gene expressing transgenic plants were developed and their tolerance under drought, salinity and oxidative stresses was studied.

## Materials and Methods

### Plant material and growth conditions

#### Finger millet

The finger millet (var. GPU 28) seeds were obtained from all India coordinated research project (small millets), University of Agricultural Sciences, GKVK Bangalore India. For the seedling level experiments, seeds were germinated on moist filter paper at 30°C and 80% relative humidity. Seedlings (1.5 to 2 cm length) were used for stress experiments. For plant level experiments, plants were grown in plastic pots with the potting mixture consisting red soil: sand: vermiculite in the ratio of 3∶1∶1 by volume (22% water holding capacity). GPU28 was used for all experiments described in this manuscript except for optimization of hormonal concentrations for callus induction and plant regeneration in MS medium.

#### Tobacco

Tobacco (var. KST 19) plants were grown in the greenhouse and ∼30–35 day old plants were used for stress experiments unless otherwise specified.

For both plant species, recommended fertilizers including micro and macronutrients were provided. Prophylactic measures were taken to maintain the plants disease and pest free. Plants were grown in the greenhouse at 22–27°C with 60% relative humidity and a 12 h photoperiod with mild-day light intensity of ∼500 µmol m^−2^ s^−1^.

### Bacterial strain and binary vectors

The binary vectors pCAMBIA 1301 having beta-glucuronidase (*UidA*) [55] and pCAMBIA 1380 having *mtlD* [52], [54] (NCBI accession ×51359), both driven by CaMV35S promoter were used in this study. Both constructs have the plant selectable antibiotic gene, hygromycin phosphotransferase II (*HptII*). These constructs were mobilized into *Agrobacterium* strain EHA105 and used for plant transformation experiments.

### *Agrobacterium*-mediated genetic transformation in finger millet

#### Callus induction

Seeds were surface sterilized with 0.1% mercuric chloride for 5 min and then rinsed for four times in sterile de-ionized water. Sterilized seeds were placed on Murashige and Skoog (MS) medium [56] with 3.0 mg/L auxin (2, 4-D) and 0.5 mg/L benzyl adenine (BA; Himedia laboratories, Mumbai, India) and incubated in dark for 30 days at 29°C. Calli obtained from the scutellum portion of the seeds were sub-cultured. Some seeds developed into seedlings and were periodically removed from the plate. Also, brown calli were discarded before sub-culturing and only embryogenic calli were used for transformation.

#### Transformation

*Agrobacterium* strains EHA105 harboring the binary constructs were grown on AB minimal medium with 50 mg/L kanamycin for 16 h at 28°C. Cultures (OD_600_ = 0.1) were resuspended in liquid co-cultivation medium supplemented with 100 µM acetosyringone (Sigma, Aldrich, India). Calli (45 day old) were incubated with bacterial (carrying *mtlD* or *UidA* gene construct) suspension medium for 10 min. They were then blotted dry on sterile filter paper to remove excess *Agrobacterium* and co-cultivated for 48 h at 25°C in dark. Subsequently, they were washed using sterile water containing 300 mg/L cefotaxime for four to five times, blotted on sterile filter paper and inoculated on antibiotic selection medium (MS+0.5 mg/L BA+3 mg/L 2, 4-D+30 mg/L hygromycin and 300 mg/L cefotaxime). This step was repeated if *Agrobacterium* growth occurred.

#### Plant regeneration

After three to four weeks of incubation the surviving calli were transferred to regeneration medium (MS with 0.5 mg/L BA) with hygromycin for plant-let growth and maintained in light for 20 days. Later, the shoot-lets were sub-cultured to rooting medium (MS with 0.5 mg/L BA and 0.1 mg/L NAA) for two weeks. Rooted plants were then transplanted in to soil mixture for further growth. Overall steps involved in mtlD transgenic plant development are described in figure S1.

### Stress imposition in finger millet seedlings

Finger millet seeds from T1 generation were germinated on moist filter paper at 30°C for 36 h and uniform seedlings were acclimated with respective stress (−0.2 MPa PEG, 50 mM NaCl, and 1 mM menadione) for 8 h. Then, the seedlings were transferred to Petri plates with filter paper containing polyethylene glycol (PEG-5000; −1.4 or −1.5 MPa) or NaCl (300 and 400 mM) or the naptho-quinone compound, menadione (Sigma chemicals, Bangalore India; 4 and 5 mM), solution. PEG, NaCl and menadione induces osmotic, salinity and oxidative stresses respectively and solutions were prepared as described in previous literatures [27], [57], [58]. Menadione undergoes rapid auto-oxidation with the regeneration of the parent quinone and thus produces superoxide radicals and hydrogen peroxide [59], [60]. After subjecting the seedlings to respective severe stress, seedlings were allowed to recover at 30°C for 72 h on the filter paper with water in the Petri plates. At the end of recovery, the root and shoot length (cm) were recorded. In all the experiments 50 seedlings were maintained per treatment. Seedlings and/or plants that are maintained at 30°C (room temperature) throughout the experimental period were considered as absolute controls [27].

### Drought stress imposition in finger millet plants

Fifteen day old plants were planted in pots filled with potting mixture of known weight. Pots were irrigated until all the soil macro and micro pores were filled and excess water was drained. Based on water holding capacity for this soil mixture, total weight of pot with soil mix for 100% field capacity (FC) was arrived. Plants were maintained at 100% FC (control) until imposition of drought stress. Drought stress was imposed by following gravimetric method as described previously [23], [61]. Briefly, acclimation was done by gradually with-holding irrigation until the soil water content reached to 50% FC over one week period. Immediately after acclimation, plants were exposed to severe stress (50% or 20% FC) by further withholding the irrigation and the plants were maintained at these stress levels for one week. At the end of the stress period, stress responses were assessed in the leaves. Soil moisture regimes were monitored by weighing the pot with plant once a day and replenished the weight lost through transpiration by adding water. Pot mixture without plant was used to know the evaporative soil water loss (blank) at the beginning of the experiment. Soil water potential was measured using WP4 dew-point potentiometer (Decagon Devices Inc, Washington, DC, USA) and the value at 100% FC was −0.03.

### Oxidative stress imposition in finger millet and tobacco plants

Leaf segments (1 cm diameter) were taken from 45 day old transgenic and wild-type tobacco or finger millet plants grown under non-stress condition. The leaf segments were initially incubated in lower concentration (0.5 µM) of methyl viologen (Sigma chemicals, Bangalore India) with 100 µmol m^−2^ s^−1^ light for 2 h for stress acclimation. They were then exposed to high light (700–800 µmol m^−2^ s^−1^) with 2 µM methyl viologen for 6 h. Methyl viologen accepts electron from the last step of photosynthetic electron transport chain and produces free radicals [62] and has been extensively used to create oxidative stress in plants and algae [55], [57], [63]. At the end of stress period the leaf segments were used for total chlorophyll estimation or cell viability estimation by 2,3,5-Triphenyltetrazolium chloride (TTC) assay. Superoxide radicals were quantified during the stress period by 2,3-bis-(2-methoxy-4-nitro-5-sulfophenyl)-2H-tetrazolium-5-carboxanilide (XTT) assay.

### Molecular confirmation of putative transgenic finger millet plants by PCR

The genomic DNA was isolated from the leaves of putative transformed and wild-type plants. The transformed plants were confirmed by PCR using primers specific to *UidA* gene 5′-GGG CAG GCC AGC GTA TCG TG-3′ and 5′-GTC CCG CTA GTG CCT TGT CCA GTT 3′ and primer pairs 5′-ATC CGC TGC CCC TCT CT-3′ and 5′-ACA TGG CCT TTC AGC TGC GTG GTA-3′ that amplify 500 bp fragment of the *mtlD* gene. Primer pairs 5′- GAG GCT ATT CGG CTA TGA CTG-3′ and 5′-ATC GCG AGG GGC GAT ACC GTA-3′ were used to amplify 750 bp fragment of *hptII* gene. Further, primer pairs 5′-TCC ATA ATG AAG TGT GAT GT-3′ and 5′-GGA CCT GAC TCG TCA TAC TC-3′ were used to amplify 300 bp fragment of *Actin* gene.

### Southern blot

Putative finger millet transgenic plants having *UidA* or *mtlD* genes were analyzed for integration and copy number of the transgene. Genomic DNA from the transgenic and wild-type plants were restrict digested (XbaI), resolved on 0.8% agarose gel and transferred to positively charged nylon membrane. The membrane was exposed to UV (1200 µJ for 60 s) for cross linking. The blot was probed with the respective radio labeled (^32^P dCTP 3000 Ci mmol) inserts. Pre-hybridization was done at 42°C for 2 h and hybridization was done at 55°C overnight with blocking solution (0.5 M Na phosphate buffer, 1 mM EDTA, and 7% SDS, pH 7.2). The hybridized blot was washed in the following sequence with 6× saline sodium citrate (SSC) for 10 min at 37°C, 4× SSC for 10 min at 37°C; 2× SSC for 10 min at 37°C; 2× SSC for 15 min at 55°C; 0.2× SSC for 10 min at 55°C. The hybridization signals were detected following exposure of Kodak X-ray film to the membrane at −70°C for 24 h and developed by autoradiography.

### Dot blot

Genomic DNA (50 ng) from finger millet plants was spotted on the nitrocellulose membrane and fixed by exposing to UV (1200 µJ for 60 s) in an UV cross linker (Herolab CL-1, Gemini BV Laboratory, The Netherlands). Probe (*Hpt*) was prepared by labeling it with ^32^P dCTP as described under Southern blot. The blot was hybridized using the protocol described under Southern blot and developed by autoradiography.

### RT-PCR

Total RNA was extracted according to the protocol described by Datta et al. (1989) and first strand cDNA was synthesized by oligo (dT) primers using Molony Murine Leukaemia Virus reverse transcriptase (MMLV-RT; MBI Fermentas, Hanover, MD, USA) according to manufacturer's instructions. The cDNA pool was used as a template to perform RT- PCR analysis using the following primers *Actin-F tccataatgaagtgtgatgt*, *Actin-R ggacctgactcgtcatactc*.and mtlD F- caagcatgcggcgtacatcc, mtlD R- gcggatcatcttcactgcggaa. PCR conditions were 94°C for 2 min, 25 cycles of 94°C for 45 s, 52–58°C for 30 s, 72°C for 30 s and a final extension of 72°C for 10 min.

### GUS histochemical assay

GUS activity in putative transformed callus was assayed as described in the literature [64]. Hygromycin resistant calli and a portion of ear head (T_0_ generation) from the PCR positive transgenic plants were incubated at 37°C in GUS staining solution [containing 50 mM phosphate buffer, pH 7.0 and 1 mM 5-Bromo 4-chloro 3-indolyl glucuronide (X-gluc; Sigma, Aldrich, India)] for 8 h. After incubation they were transferred to 80% (v/v) ethanol/water solution to remove the chlorophyll, and later photographed. Similarly, seedlings (T_1_ generation) were stained with GUS solution and photographed.

### Quantification of osmotic adjustment

From the drought exposed (gravimetrically) finger millet plants 5^th^ leaf was excised from stressed and well-watered plants and sampled at midday, quickly sealed in plastic bags, and kept on ice. These leaves were used to determine the relative water content (RWC) and solute potential.

#### Relative water content

After determining the fresh weight, the leaf segments were floated on deionized water for 5 h to determine their turgid weight. The dry weight was determined after oven-drying to a constant weight. The RWC was calculated using the formula:
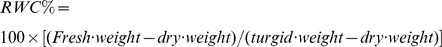



#### Solute potential of cells in leaf (Ψs)

Leaf samples were frozen in liquid nitrogen, thawed, and centrifuged for 5 min at 20 000 g. The Ψs of the extracted sap was measured by VAPRO vapor pressure osmometer (Wescor Inc., Logan, UT, USA). 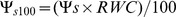



#### Osmotic adjustment

From the values of RWC and solute potential of control and stress-grown plants, the osmotic adjustment was calculated using the formula: Osmotic adjustment (OA)  =  drought leaf Ψ_S100_ - control leaf Ψ_S100_

### Estimation of chlorophyll content

Chlorophyll was extracted from 100 mg of leaf tissue in 1 ml of acetone and dimethyl sulphoxide (1∶1, volume/volume) mix. The absorbance was recorded at 663 and 645 nm using UV–visible spectrophotometer Model DU800 (Shimadzu Corporation, Kyoto, Japan). Total chlorophyll was estimated as described previously [65] and expressed as percent reduction relative to the corresponding control.

### Mannitol estimation

The standard graph was developed using Mannitol (Himedia laboratories, Mumbai, India) using the previously described method [43], [66] with below mentioned modifications. Surface-sterilized seeds were ground in ethanol and centrifuged at 7000 g for 5 min at room temperature. Similarly the surface sterilized germinating seedlings was ground in ethanol and centrifuged. Mannitol (Sigma Aldrich, Bangalore, India) chemical was used to prepare standard. Distilled water (2 ml) was used as reagent blanks. One blank (oxidized) was carried through the procedure as described below. The other (unoxidized) was treated with stannous chloride (Himedia laboratories, Mumbai, India) prior to addition of periodic acid. Periodic acid reagent (Himedia laboratories, Mumbai, India) (0.5 ml) was added to the oxidized blank and sample tubes (2 ml of supernatant). Later, the contents were mixed and incubated at room temperature for 8 to 10 min. To these tubes 0.5 ml of stannous chloride was added. The stannous chloride is known to be oxidized by periodate into stannic acid, which appears as a milky precipitate in the tube. In order to solubilize the precipitate, 5 ml of chromotropic acid reagent was added rapidly and the mix was vigorously vortexed. The tube was placed in a boiling water bath for 30 min and then cooled and volume was made up to 25 ml with distilled water. Absorbance at 570 nm was measured using spectrophotometer [67] (Spectramax plus, Spinco Bioteck, Bangalore India).

### TTC assay

Cell viability was measured by 2,-3,-5-triphenyltetrazolium chloride (TTC; Sigma, Aldrich, India) as described in the previous literature [68]. Briefly, TTC assay solution (0.1%) was prepared by dissolving TTC in sodium phosphate buffer (pH 7.4). Segments (1 cm diameter each having dry weight 0.27 mg) from stressed and control finger millet leaves were initially washed in sterile water and incubated in the TTC solution at room temperature for 5 h in dark. Subsequently, they were boiled with 5 ml of 2-methoxy ethanol until dryness to extract bound formazan. To this 5 ml of 2-methoxy ethanol was again added and absorbance was measured at 485 nm using UV–visible spectrophotometer Model DU800.

### XTT assay

Leaf segments (1 cm diameter each having dry weight (0.27 mg) were incubated in 1 ml of K-phosphate buffer (20 mM, pH 6.0) containing 500 µM of XTT (cell proliferation kit II, Roche Diagnostics Corporation, Basel, Switzerland) along with methyl viologen and exposed to high light stress. XTT is a sodium 3′[1-(phenylamine-carbonyl-3, 4-tetrazolium)-bis [4 methoxy-6-nitro] benzene sulfonic acid hydrate]. Later, increase in absorbance at 470 nm of incubation medium was measured using a micro titer plate reader (Sunrise-Magellan, Tecan, Australia) as described in the previous literature [69].

### Statistical analysis

Analysis of variance (anova) was performed using MS Excel software, and data points were analyzed for Fisher's least significant difference (LSD, *P*≤0.05) or Duncan's multiple range test or students *t*-test. Statistical significance of values in graphs was indicated either as asterisks or as letters.

## Results

### Callus induction and plant regeneration in finger millet

**Figure 1:**
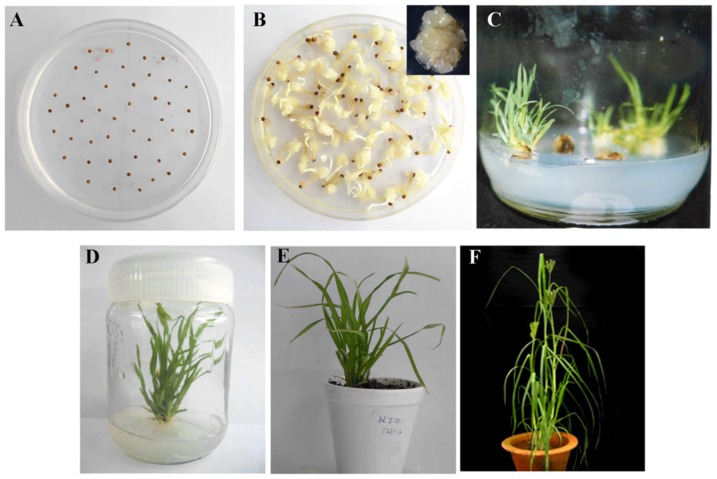
Callus induction and regeneration from scutellum portion of matured seeds in finger millet. Seeds were incubated on the callus induction medium for 30 days (A), and the calli developed from scutellum portion of seeds was photographed (B). Further, these calli were transferred to regeneration medium and fifteen days later shoot initiation was photographed (C). Later, these shoot-lets were transferred to root induction medium (D). After this step, plants were transferred to potting mixture for hardening (E) and were transferred to greenhouse. Inset in (B) shows enlarged callus derived from single seed.

Fully matured finger millet (var. GPU28) seeds were used as explant (Figure 1A). Among 11 different hormonal treatments two of them namely, MS medium containing 0.5 mg/L BA & 2 mg/L 2, 4-D and MS medium containing 0.5 mg/L BA & 3 mg/L 2, 4-D showed higher callus growth ranging from 200–245 mg/seed callus (Table S1). MS medium with 0.5 mg/L BA & 3 mg/L 2, 4-D eventually produced better quality embryogenic calli. Hence, this concentration was used for callus induction in this experiment. Seven days after incubation of sterilized seeds in callus induction medium, callus initiation was observed from the scutellum portion of the seeds. After sub-culturing two times in ∼15 days, embryogenic calli were observed indicating the hormone concentrations used in callus induction medium was successful for callus induction (Figure 1B). Further, to achieve efficient regeneration of shoot-lets, ∼30 day old hard calli were sub-cultured on to regeneration medium. Hormonal concentration for optimum regeneration of shoots was initially optimized and MS medium with 0.5 mg/L BA was found to produce green and good quality shoot-lets (Table S2). Results showed that hard friable calli produced better morphogenesis and shoot-lets than the soft watery calli. Green tissues and subsequent initiation of shoots from theses calli was seen after 15 days. Later, these shoot-lets were sub-cultured and multiple shoots were obtained (Figure 1C). Further, individual shoots were transferred to fresh regeneration medium for root initiation (Figure 1D) and plants were allowed to grow till 4–5 leaves stage. MS medium with 0.5 mg/L BA and 0.1 mg/L NAA provided better rooting compared to MS medium with 0.5 mg/L BA. Plants were then transferred to styrofoam disposable cups having sterile vermiculite, covered with polythene bags and maintained in growth room for three weeks (Figure 1E). Later, hardened plants were transferred to soil mixture and grown in greenhouse (Figure 1F). These plants flowered and produced seeds normally, demonstrating the successful generation of finger millet plants from scutellum derived calli.

### Development of transgenic finger millet expressing *UidA* gene through *Agrobacterium*-mediated transformation

**Figure 2:**
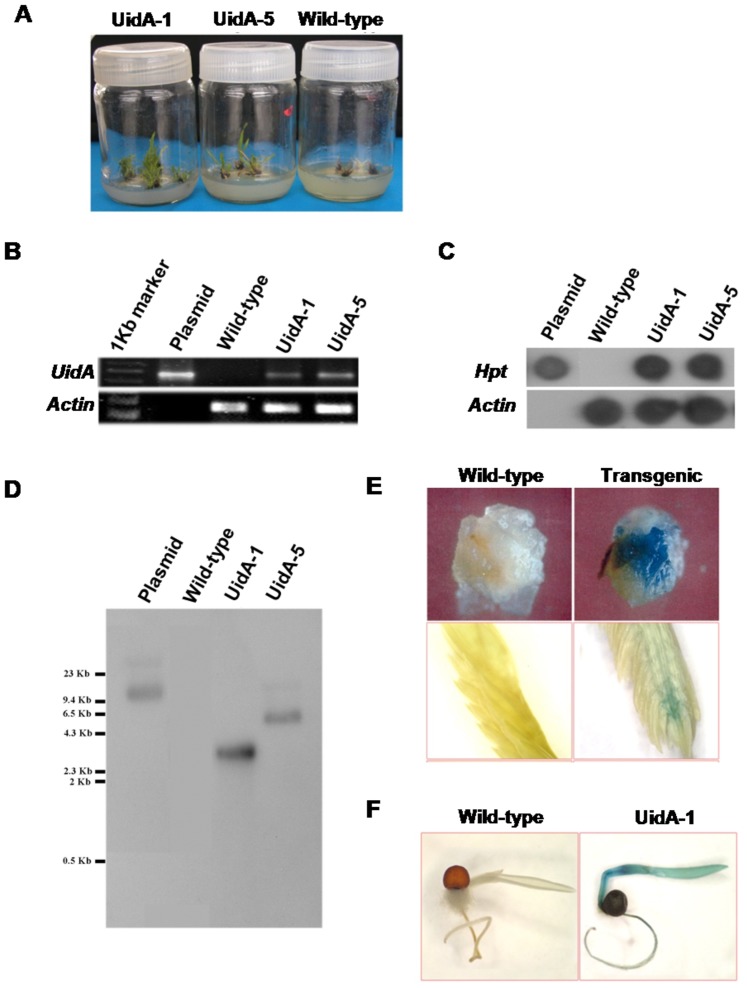
*Agrobacterium*-mediated *UidA* gene transformation in finger millet. Initially calli were infected with *Agrobacterium* harboring *UidA* gene construct and co-cultivated for two days and transferred to the hygromycin selection medium. Photograph of survived calli on selection medium was taken after 30 days (A). Plants (T_0_) obtained from the survived calli were analyzed for the presence of *UidA* gene by PCR (B) and for *HptII* gene by genomic dot blot (C). Also, the copy number of *UidA* gene was assessed by Southern blot (D). GUS histochemical assay was performed in transformed calli (T_0_ generation; upper panel), T_0_ generation finger-let from ear head (UidA-1 T_0_; lower panel) (E) and 3 day old T_1_ generation seedlings (UidA-1) and the GUS expression were photographed (F). UidA-1 and UidA-2 indicate two independent transgenic lines.

Embryogenic calli infected with *Agrobacterium* strain EHA105 harboring pCAMBIA 1301/*UidA* constructs were co-cultivated for 48 h. The *Agrobacterium* culture was incubated with 100 µM of acetosyringone for virulence gene induction [70]. Previously standardized hygromycin concentration (30 mg/L) was used for selecting the transformed calli [71], [72]. After co-cultivation, the infected calli were incubated on the selection medium (MS+0.5 mg/L BA+3 mg/L 2, 4-D+30 mg/L hygromycin and 300 mg/L cefotaxime) under light (100 µmol m^−2^ s^−1^) for 10 days. The surviving calli were then transferred to regeneration medium (MS+0.5 mg/L BA+30 mg/L hygromycin and 300 mg/L cefotaxime). Later, as per previously standardized protocol (Figure 1C), they were sub-cultured into shooting medium with hygromycin (Figure 2A). Hygromycin resistant independent shoots were developed in to plants.

PCR analysis using *UidA* and *HptII* gene specific primers showed presence of *UidA* gene in putative transgenic plants (T_0_ generation; Figure 2B). Further, the genomic dot blot analysis using *HptII* gene specific probe showed the signals in the lane loaded with DNA from transgenic lines and no signal was observed in the wild-type (Figure 2C) suggesting the integration of construct. To identify the copy number of *UidA* gene, Southern blot was performed using *UidA* gene specific probe. The genomic DNA was digested with *Xba*I which cuts once in T-DNA. The hybridization signals in blot for the two independent transgenic lines (UidA-1 and UidA-5) showed single copy integration of *UidA* gene in the finger millet genome (Figure 2D).

### GUS expression

To further confirm *UidA* gene expression at protein level, GUS-histochemical staining in callus and a portion of ear head was performed. The transgenic callus stained blue with X-gluc indicating the GUS activity, whereas wild-type callus did not stain (Figure 2E). Similarly, ear head from the transformed line (UidA-1) showed intense staining (Figure 2E). Further, 3 day old seedlings (T_1_ generation line, UidA-1) selected on the hygromycin (Figure S2) were also subjected to histochemical staining. Dark blue staining of shoots, young leaves and roots further confirmed the stability of the transgene expression in T_1_ generation (Figure 2F).

Further, transformation efficiency was computed based on the number of calli infected and number of calli regenerated into shoots in the selection medium. Results showed an average of 6% transformation efficiency (Table S3). Taken together, these results demonstrated the establishment of regeneration and *Agrobacterium*-mediated transformation protocols in finger millet.

### Development of transgenic finger millet plants expressing *mtlD* gene by *Agrobacterium*-mediated transformation

**Figure 3:**
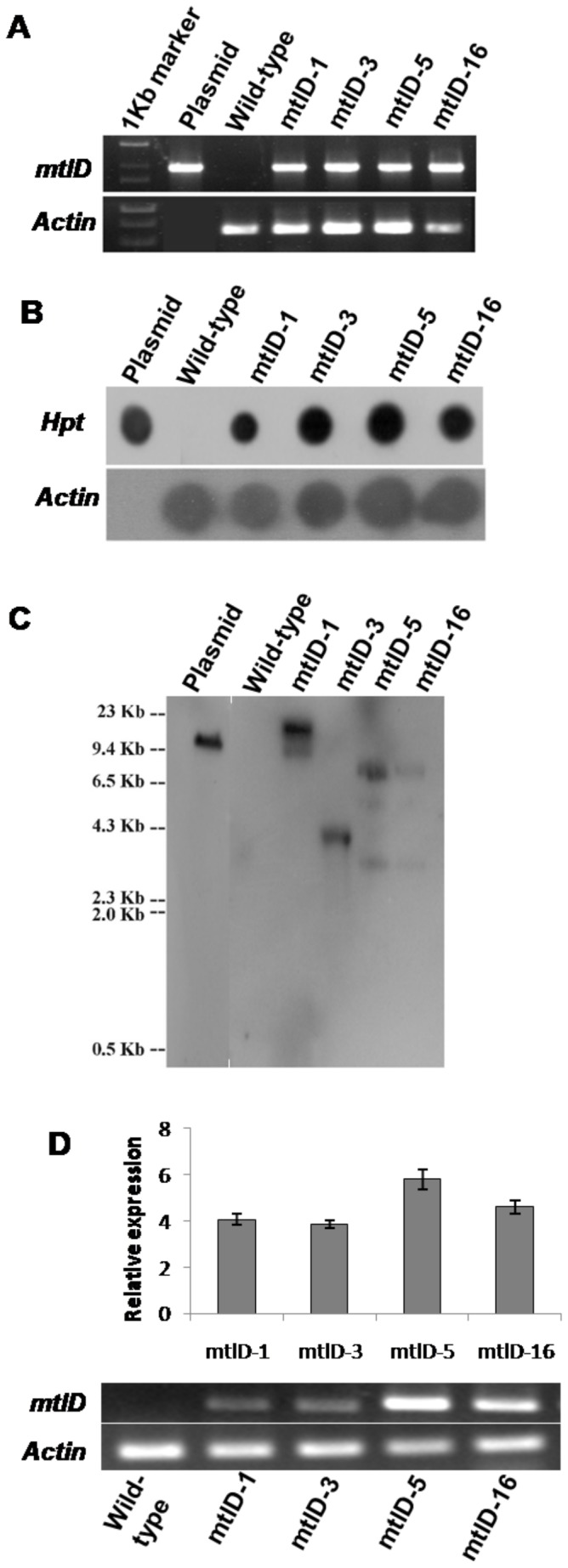
Molecular characterization of finger millet transgenic plants expressing *mtlD* gene. Putative finger millet transgenic lines (T_0_ generation) transformed with pCAMBIA 1380-mtlD construct were analyzed for the presence of *mtlD* gene by PCR (A) and *HptII* gene by dot blot analysis (B). Also, Southern blot was performed to assess the copy number of *mtlD* gene in these transgenic plants (C). Transcript expression of transgene was analyzed in transgenic plants by semi quantitative RT-PCR. Quantity one (Biorad) software was used to measure the band intensity. The corresponding increase in the relative density of bands over wild-type is presented in the histogram (D).

With the aim to improve drought, oxidative and salinity tolerance in finger millet during early seedling growth, the pCAMBIA 1380/mtlD construct was used for mannitol production in finger millet. Adapting the regeneration and transformation protocols developed in this study, the finger millet (var. GPU28) putative transgenic plants expressing mtlD were developed. Hygromycin resistant putative transgenic finger millet seedlings (Figure S3) from independent transgenic events were analyzed by PCR, genomic dot blot and Southern blot. PCR analysis using the *mtlD* gene specific primers showed amplification in all the selected putative transgenic plants whereas no amplification was observed in wild-type plants (Figure 3A). Further, genomic dot blot analysis also confirmed the presence of *HptII* gene in the transgenic lines (Figure 3B). Further, copy number of *mtlD* gene integrated in the genome was assessed by Southern blot (Figure 3C). The expression of *mtlD* gene in fingermillet was confirmed by RT-PCR (Figure 3D). The segregation analysis [73] revealed that the transgenic events are likely to have monogenic inheritance (Table 1). Unlike reported in a previous study [74] where wheat plants accumulating higher mannitol showed stunted growth, finger millet transgenic plants showed normal growth. For example, 15 day old wild-type and transgenic plants maintained ∼10 leaves per plant with similar leaf size and overall plant height.

**Table 1:** Segregation analysis for mtlD finger millet transgenic seedlings.

	Number of seedlings incubated	Number of seedlings survived*	Chi square value**
mtlD-1	71	53	(3∶1) 0.731429
mtlD-3	77	51	(3∶1) 1.958748
mtlD-5	68	50	(3∶1) 0.732168
mtlD-16	71	50	(3∶1) 1.047471

*one day old transgenic (T_1_ generation) seedlings were incubated on 60 mg/L of hygromycin. 5 days after incubation they were scored for hygromycin resistance based on their survival.

**The chi-square test was performed using MS Excel POPTOOLS.

### Performance of *mtlD* expressing transgenic finger millet plants under methyl viologen-induced oxidative stress

**Figure 4:**
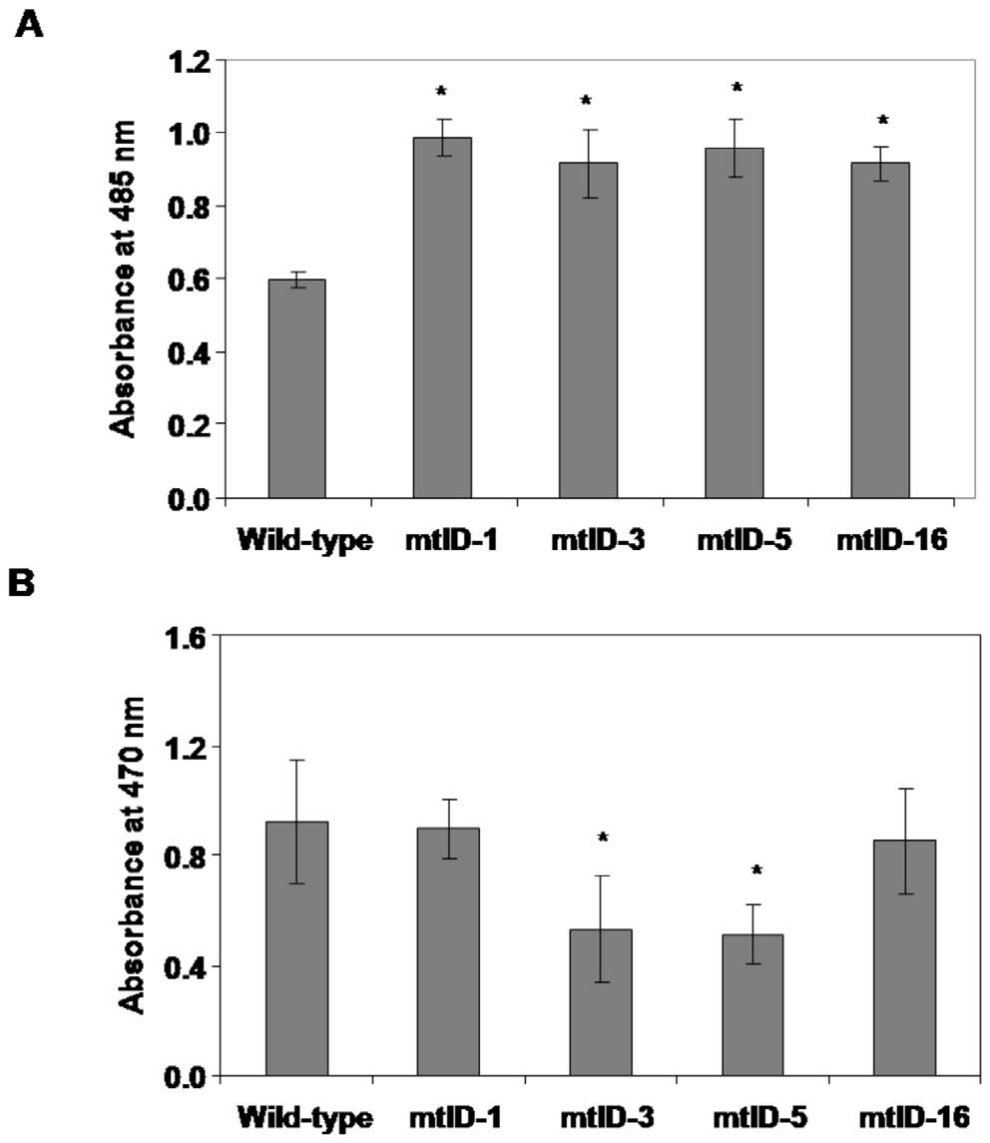
Cell viability and superoxide radical accumulation in the finger millet transgenic plants expressing *mtlD* gene under methyl viologen coupled with high light-induced oxidative stress. Excised leaf segments from T_1_ generation finger millet transgenic plants were exposed to oxidative stress as described in material and methods section. At the end of stress, a sub set of leaf segments were incubated in TTC solution and cell viability was calorimetrically estimated by measuring the extent of reduction of TTC into formazan (A). Another sub set of leaf segments were used for superoxide radical estimation during stress. XTT solution was added to the incubation medium and XTT reduction during stress was measured by calorimetric method (B). Each bar represents the mean of standard error values (n = 10). Experiments were repeated twice. Result from one experiment is presented here and results for another independent experiment were similar. Asterisks indicate values are statistically significant (student's t test; *p*<0.05) versus corresponding wild-type.

Drought stress and salinity stress induces higher levels of free radical production in finger millet [16]. Mannitol is known to reduce injury to the plants from ROS generated during drought and salinity stress [36], [74], [75]. Hence, in order to select stress tolerant plants among the putative transgenic plants methyl viologen-induced oxidative stress was imposed. At the end of stress period cell viability (TTC assay) was assessed. Transgenic lines maintained higher cell viability compared to wild-type plants under MV stress (Figure 4A). During the stress superoxide radical production was estimated by XTT assay. The transgenic lines mtlD-3 and mtlD-5 showed lower levels of XTT reduction compared to wild-type indicating lower levels of superoxide radicals in the transgenic lines (Figure 4B). Seeds from mtlD-1, mtlD-3 and mtlD-5 lines were used for further experiments.

### Assessing the drought stress tolerance of mtlD expressing transgenic finger millet plants

Transgenic seedlings were subjected to PEG, NaCl and menadione stress and their growth were measured. Transgenic seedlings from all three lines (mtlD-1 T_1_, mtlD-3 T_1_ and mtlD-5 T_1_) showed moderate level of stress tolerance under PEG-induced osmotic stress (Figure S4A). However, only seedlings from mtlD-1 T_1_ showed increased salinity tolerance compared to wild-type (Figure S4B). Also, only the mtlD-5 T_1_ seedlings showed increased growth under oxidative stress compared to wild-type (Figure S4C).

**Figure 5:**
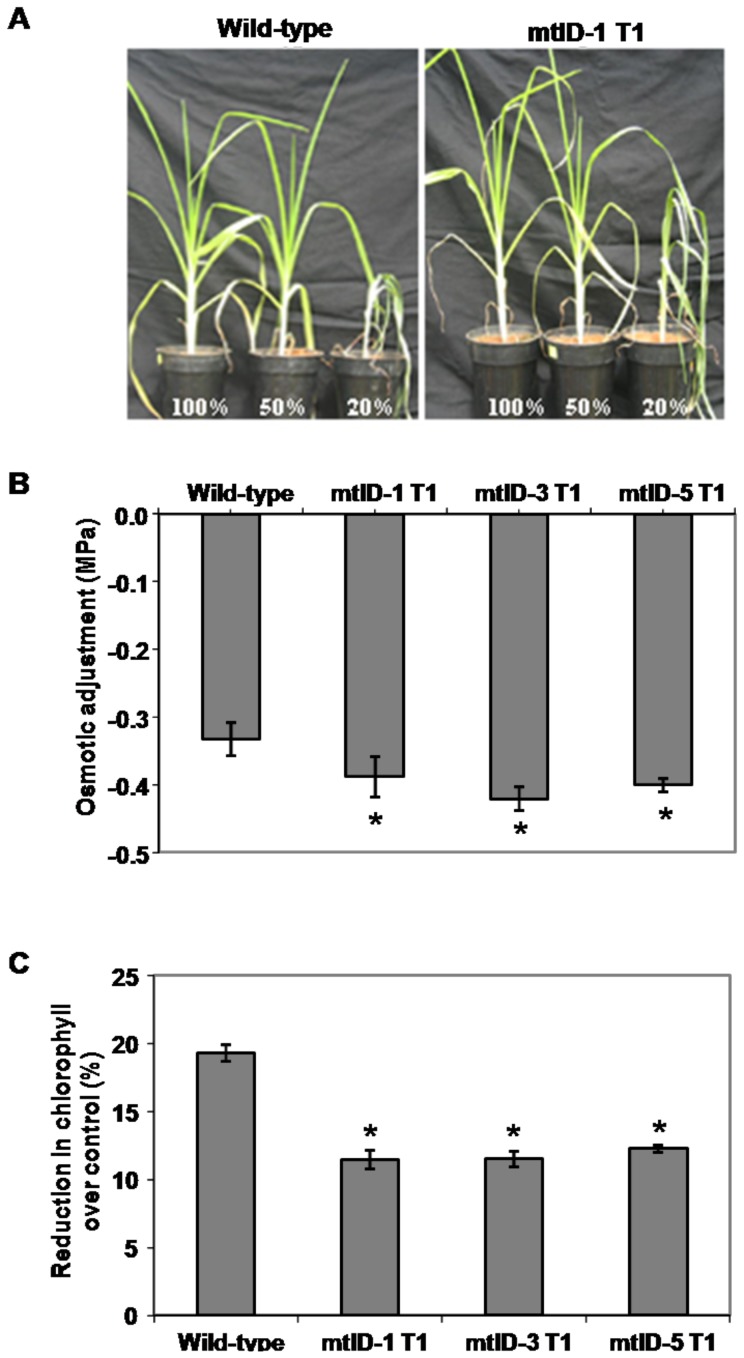
Effect of drought stress on the growth, osmotic adjustment and chlorophyll content of *mtlD* transgenic finger millet plants. Drought stress was imposed to 15 day old plants (T_1_ generation) by gradually withholding irrigation in such a way that the soil moisture reaches to 50% FC in one week. Later, plants were exposed to severe stress (50% or 20% FC) for 7 days. Photographs (line mtlD-1 T_1_) were taken at the end of the stress (A). Stress tolerance of the transgenic plants exposed to 50% FC was analyzed by measuring osmotic adjustment (B) and percent reduction in chlorophyll over respective controls (C). Each bar represents the mean of standard error values (n = 5). Asterisks indicate values are statistically significant (student's t test; *p*<0.05) versus corresponding wild-type. Values mentioned in the picture represent respective filed capacity.

Further, transgenic plants (T_1_ generation) were grown for 15 days in pots and used for testing their drought tolerance. Initially the plants were subjected to gradual acclimation stress and later maintained at severe stress levels for seven days. Phenotypic observations were made from the stressed plants (mtlD-1 T_1_) and wild-type. At 50% FC stress for seven days, wild-type and the transgenic plants did not show visible wilting phenotype. At much higher stress levels (20% FC) the transgenic plants showed improved tolerance to drought stress than wild-type plants (Figure 5A). Stressed plants reduced relative water content compared to non-stressed plants (Figure S4D). Transgenic plants (mtlD-1, mtlD-3 T_1_ and mtlD-5 T_1_) exposed to 50% FC stress maintained higher osmotic adjustment and ∼10% less chlorophyll degradation in all the three transgenic lines than wild-type plants (Figure 5B and 5C).

### Assessing the osmotic, salinity and oxidative stress tolerance of finger millet *mtlD* transgenic seedlings

**Figure 6:**
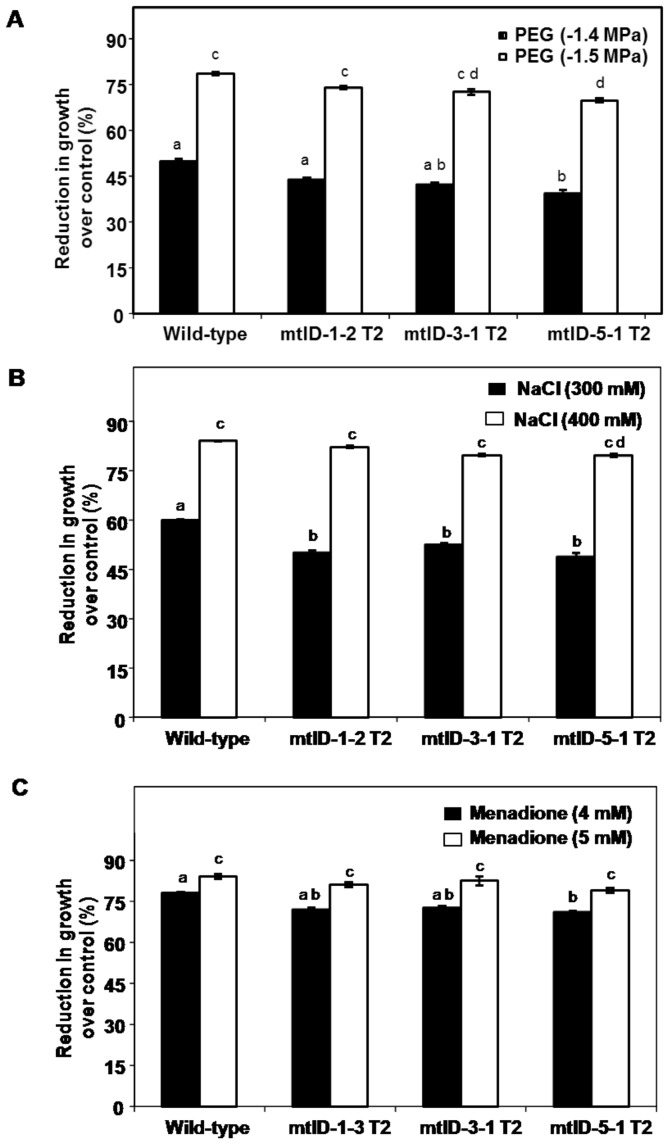
Performance of transgenic finger millet seedlings expressing *mtlD* gene under osmotic, salinity and oxidative stress. Finger millet transgenic (T_2_ generation) and wild-type seedlings (1.5 cm length) were initially acclimated with lower concentration of corresponding stress (−0.2 MPa PEG, 50 mM NaCl, and 1 mM menadione) for 8 h and further subjected to indicated concentrations of respective severe stress levels for 48 h. Seedlings were allowed to recover for two days and recovery growth after osmotic stress (A), salinity stress (B) and menadione induced oxidative stress (C) were measured and percent reduction in growth over corresponding control was calculated. Each bar represents the mean of standard error values (n = 20). Experiments were repeated twice. Result from one experiment is presented here and results for another independent experiment were similar. Alphabets above bar indicates the statistical significance (ANNOVA). Same alphabets indicate no significant difference (*p*<0.05).

**Figure 7:**
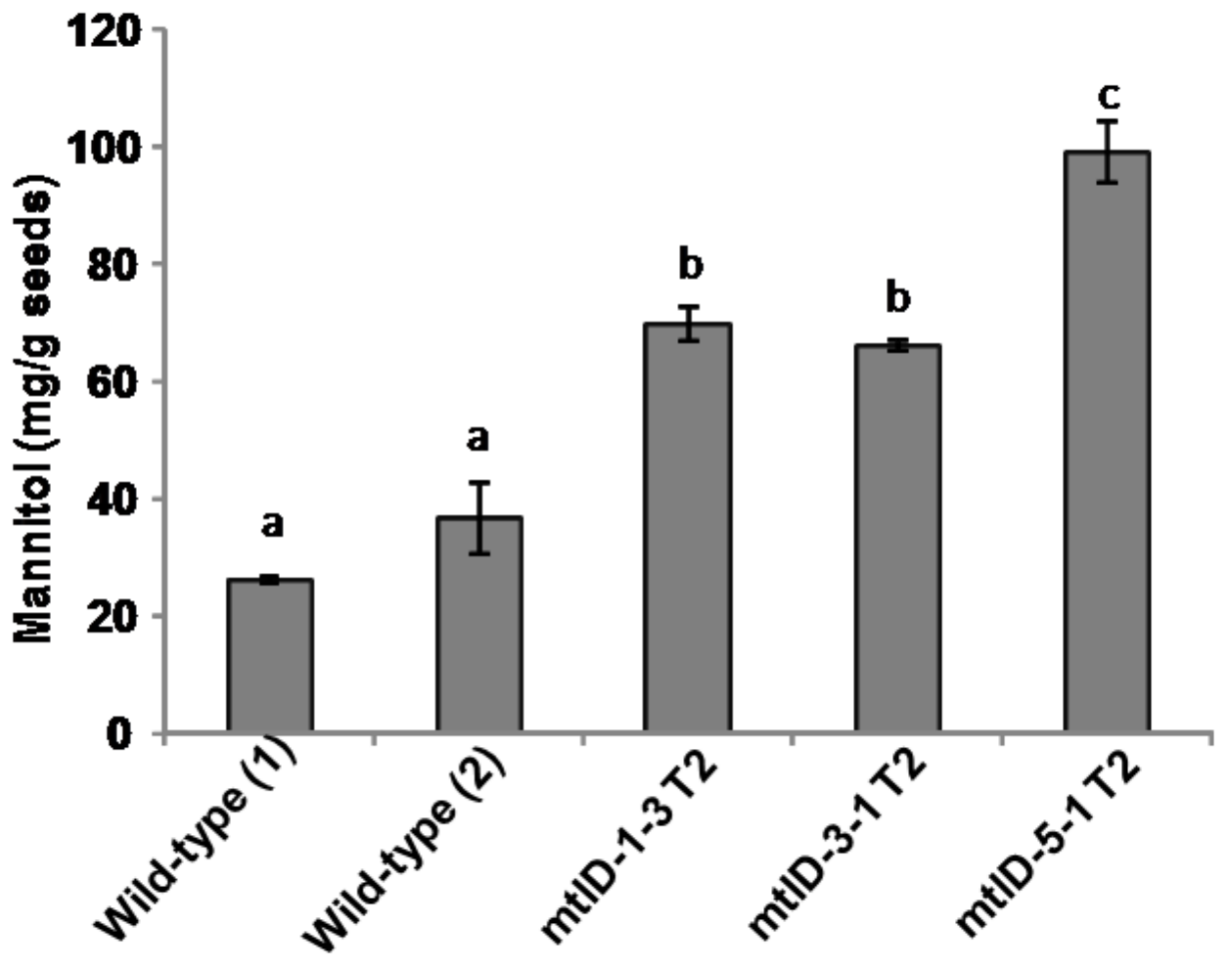
Mannitol accumulation in *mtlD* gene expressing finger millet. Mannitol content in the seeds obtained from T_2_ generation plants were estimated for the transgenic lines. Alphabets above bar indicates the statistical significance (Duncan's multiple range test). Same alphabets indicate no significant difference (*p*<0.05). Experiments were repeated twice. Result from one experiment is presented here and results for another independent experiment were similar. WT (1) and WT (2) are two independent replicate plant seeds.

The T_2_ generation transgenic seedlings (1.5–2.0 cm length) germinated on hygromycin medium (Figure S5) were transferred to PEG or NaCl or menadione stress and recovery growth was assessed. Transgenic and wild-type seedlings exposed to PEG, NaCl and menadione showed growth reduction compared to their no-stress control. However, mtlD-1-2 T_2_ seedlings showed no significant improvement in stress tolerance under PEG stress (Figure 6A). Similarly, mtlD-5-1 T_2_ seedlings showed only slight improvement in growth under PEG-induced osmotic stress (Figure 6A). The transgenic seedlings exposed to 300 mM NaCl showed slight improvement in growth compared to wild-type. However, at 400 mM the growth differences of transgenic seedlings were not significantly different from wild-type (Figure 6B). Stress tolerance of all the three lines was equal to wild-type under menadione-induced oxidative stress (Figure 6C). In order to ensure that these transgenic seedlings indeed accumulated mannitol, seeds obtained from transgenic plants (T_2_ generation) were used for mannitol quantification. Results showed that mannitol content was high in all the tested seed lots (Figure 7). Similarly the mannitol content was higher in germinated seedlings of transgenics (Figure S6). This calorimetric assay also detected ∼30 mg of mannitol per gram of wild-type seeds. This indicates finger millet may naturally accumulate mannitol in seeds. However, at this time, we do not have experimental evidence for the pathway involved in mannitol synthesis in finger millet. Also, BLAST search for identifying homologs of mannitol biosynthetic genes of celery in foxtail millet, phylogenetically close plant species to finger millet, did not reveal any hits. Nevertheless, our results indicated that mannitol accumulation has marginally improved stress tolerance in finger millet.

In order to understand the reason for only marginal improvement in oxidative stress tolerance levels in transgenic finger millet, transgenic tobacco plants expressing mtlD was used and compared their stress tolerance increase with finger millet plants (mtlD-5 T_1_). Wild-type tobacco plants are more susceptible to stress compared to wild-type finger millet [76]. The stress tolerance of tobacco and finger millet transgenic plants expressing mtlD was analyzed by subjecting the leaf segments to methyl viologen coupled with high light-induced oxidative stress. The transgenic tobacco plants showed ∼10% increase in chlorophyll maintenance under stress compared to its wild-type (Figure S7). However, the finger millet transgenic plants showed only ∼4% increase in chlorophyll maintenance compared to wild-type plants (Figure S7).

## Discussion

Genetic engineering of finger millet for improved abiotic stress tolerance is stymied because of the lack of an efficient method for genetic transformation. Here we discuss development of plant regeneration from scutellum derived embryogenic callus and also a reproducible protocol for genetic transformation by *Agrobacterium*-mediated method.

Callus induction and plant regeneration in finger millet has been reported previously using shoot apex [77]–[80]. In this study scutellum derived callus production was standardized. This protocol has two major advantages. First, scutellum derived callus possess higher regeneration efficiency compared to callus induction from apical meristem. Second, since this protocol uses seeds as explants, limitation due to explant generation during large scale studies can be overcome. For example, seed can be directly used for callus induction, whereas, for obtaining shoot apex explant at least few additional weeks are needed for growing sterile seedlings from seeds [17], [32]. Despite these studies showing callus induction and regeneration, only a few studies have demonstrated genetic engineering of finger millet for agronomically important traits. Recent preliminary studies in our lab that were aimed to develop transgenic finger millet plants for improved grain zinc content [81]and salinity stress tolerance [82] using the method developed in this study demonstrated reproducibility of the method described in this study.

Particle gun-mediated genetic transformation has been one of the methods used for transforming finger millet [17], [32]. However, this method is expensive and usually results in multiple insertions of the transgene in the genome. Multiple insertions negatively impact both laboratory research and commercial release of transgenic plants [83]. Contrary to particle gun-mediated method, *Agrobacterium*-mediated transformation is simple, permits large scale experiments with less cost and has higher reproducibility. Hence, protocol described in this study and two other recent studies [31], [84] used *Agrobacterium*-mediated transformation. These transformation protocols are expected to widen the scope for genetic engineering in finger millet in future (Figure S8).

By using the *Agrobacterium*-mediated method finger millet plants expressing *mtlD* gene were developed. The transgenic plants exhibited tolerance under drought, salinity and oxidative stress. The transgenic plants marginally performed better compared to the corresponding wild-type plants under both drought and salinity stress. For example, *mtlD* gene expressing transgenic plants showed enhanced osmotic adjustment under drought stress and also transgenic seedlings showed better growth under osmotic stress and salinity. It is possible that mannitol accumulated in finger millet transgenic plants acted as osmoprotectant and protected cells from free radicals in addition to inducing several other stress tolerance mechanisms as inferred in previous studies [36], [40], [53], [74] and hence leading to increase in their tolerance compared to wild-type. However, the fold improvement in finger millet (a C4 plant) stress tolerance, especially the oxidative stress tolerance, compared to wild-type was much lesser than the increase in tolerance of several C3 plants expressing *mtlD* gene under several abiotic stresses (Table S4). For example, increase in seedling growth in eggplants expressing *mtlD* gene was up to 80% over their wild-type under both PEG and NaCl stress (Table S4). However, finger millet transgenic seedlings showed only an average increase of ∼10% over their wild-type. Consistently, transgenic sorghum (C4) plants expressing *mtlD* gene showed only up to 23% increase in biomass under salinity stress compared their wild-type plants [53], whereas, C3 plants like rice and wheat transgenic plants showed an average of 80% and 50% increase over their corresponding wild-type [54], [74].

In order to confirm these observations that are based on previous literature information, we developed tobacco plants expressing *mtlD* gene and analyzed their performance under oxidative stress. Interestingly the tobacco transgenic plants showed higher level (∼13%) of chlorophyll maintenance over wild-type under oxidative stress (Figure S7). However, finger millet transgenic seedlings expressing *mtlD* gene showed only slight improvement (<3%) in oxidative stress tolerance (Figure 6). Similarly, at plant level, increase in chlorophyll stability in *mtlD* finger millet lines over wild-type was only 7%. This indicated that *mtlD* gene expression in finger millet imparted only marginal level of tolerance over wild-type. Consistent with these results the ascorbate peroxidase gene, *EcApx1*, cloned from finger millet has been attributed to impart better oxidative stress tolerance in the wild-type finger millet [85]. Tobacco plants expressing a NAC class of transcription factor, *EcNAC1*, from finger millet showed better chlorophyll stability under oxidative and salinity stresses [63]. Also, finger millet [86] and maize have been shown to maintain higher membrane stability and also overall plant growth under oxidative stress compared to tomato and beans [87]. Taken together, the tolerance levels of finger millet mtlD expressing transgenic plants are only marginal because wild-type plants may have higher intrinsic basal tolerance [63]. Hence, we speculate that an additional scavenging system brought out by mannitol have not dramatically contributed for stress protection in transgenic plants over wild-type.

The mechanism for only marginal increase in stress tolerance of finger millet could be due to mannitol-mediated osmotic regulation and free radical scavenging [88]. Although mannitol accumulated in finger millet mtlD expressing transgenic plants have contributed to cellular protection through its osmolytic properties, high levels of endogenous accumulation of proline [49], [89] in both wild-type and mtlD expressing transgenic plants countered the net contribution due to mannitol alone. Similarly, the germinating seeds also showed higher accumulation of mannitol (figure S6) indicating contribution of mannitol biosynthesis in reducing the free radical generation. In the transgenic plants also the scavenging ability have not been high because the wild-type plants already has efficient free radical scavenging ability [85]. It is also possible that wild-type finger millet plants better managed oxidative stress compared to tobacco because of their unique adaptation. For example, finger millet has better re-positioning of chloroplast when exposed light stress compared to tobacco [90]. Faster relocation of chloroplasts away from the high light exposed regions protects photosynthetic machinery in C4 species, finger millet [90]. However, the exact mechanism of mtlD-mediated mannitol production in plants and the fate of mannitol produced, including its internal transport during stress [91], is not yet understood.

## Conclusions

Regeneration and *Agrobacterium*-mediated transformation protocols in finger millet are demonstrated in this study. Finger millet genetic transformation described in previous study using shoot apex as explant [84] and this study are useful for this crop improvement. Salient features of this study include (1) use of commercially important finger millet (var. GPU28); (2) efficient explant type (mature seeds). Our results also reiterate the earlier notion that finger millet may have better adaptation to abiotic stress. Hence, other novel strategies should be developed for further improving stress tolerance in finger millet.

## Supporting Information

Figure S1 **Steps involved in development of finger millet transgenic plants expressing** ***mtlD*** **gene.** The T_0_ transgenic plants (var. GPU28) which were confirmed by PCR and Southern blot were grown in greenhouse and seeds were collected. For further experiments, out of 20 putative transgenic events, four T_0_ transgenic events were used. These four events were designated as mtlD-1, mtlD-3, mtlD-5, mtlD-16 and taken forward. Seeds obtained from three (T_0_) events (mtlD-1, mtlD-3 and mtlD-5) were used for further experiments. Seeds from each event were bulked and used for seedling experiment (named as mtlD-1 T_1_, mtlD-3 T_1_ and mtlD-5 T_1_) or plant level experiment. Plants obtained from individual seeds representing each (T_1_) event were assessed for presence of *mtlD* gene by PCR and then seeds were collected from these plants. These seeds were designated as mtlD-1-2 T_2_, mtlD-3-1 T_2_ and mtlD-5-1 T_2_ and used for mannitol quantification. A sub-set of seedlings obtained from these seeds were used for stress experiments.
(PDF)

Figure S2 **Germination of seeds obtained from** ***UidA*** **gene expressing finger millet transgenic plants on hygromycin.** Wild-type (var. GPU28) finger millet seeds were germinated in petri dishes on filter paper with different concentration of hygromycin for 5 days at 30°C with 70% relative humidity in dark and germination percentage was recorded at the end of treatment period (A). Similarly, seeds obtained from the GUS positive transgenic finger millet plants (UidA-1; T1 generation) and wild-type (var. GPU28) were germinated on hygromycin (60 mg/L) and number of seedlings survived was recorded (B). Asterisks indicate values are statistically significant (student's t test; *p*<0.05) versus corresponding wild-type. Each bar represents the mean of standard error values (n = 10).
(PDF)

Figure S3 **Growth of mtlD expressing finger millet transgenic (T_1_ generation) and wild-type seedlings on hygromycin medium.** Seeds from mtlD transgenic finger millet (T1; mtlD-1 T1 generation) and wild-type (WT, var. GPU28) were inoculated on the MS medium containing hygromycin (20 mg/L or 40 mg/L) for five days. Seedling growth on antibiotic medium was photographed at the end of treatment period.
(PDF)

Figure S4 **Performance of mtlD expressing finger millet transgenic seedlings under osmotic, salinity and oxidative stress.** Finger millet transgenic (T_1_ generation) and wild-type (var. GPU28) seedlings (1.5 cm length) were initially acclimated with lower concentration of corresponding stresses (−0.2 MPa PEG, 50 mM NaCl, and 1 mM menadione) for 8 h and then subjected to indicated concentrations of respective severe stress levels for 48 h. Seedlings were allowed to recover for two days and recovery growth of osmotic stress (A), salinity stress (B) and menadione induced oxidative stress (C) were measured and percent reduction in growth over corresponding control was calculated. The percent relative water content was measured from the plants exposed to 100%, 50% and 30% field capacity (D). Each bar represents the mean of standard error values (n = 20). Experiments were repeated twice. Alphabets above bar indicates the statistical significance (ANNOVA). Same alphabets indicate no significant difference (*p*<0.05).
(PDF)

Figure S5 **Growth of mtlD expressing finger millet transgenic (T_2_ generation) and wild-type seedlings on hygromycin medium.** Seeds from mtlD expressing transgenic finger millet (mtlD-1-2 T_2_) and wild-type (WT, var. GPU28) were incubated on the MS medium containing hygromycin (20 mg/L or 40 mg/L) for five days. Seedling growth on antibiotic medium was photographed at the end of treatment period.
(PDF)

Figure S6 **Mannitol accumulation in** ***mtlD*** **gene expressing finger millet germinating seedlings.** Mannitol content in the seedlings obtained from T_2_ generation plants were estimated as described in material and methods. Alphabets above bar indicates the statistical significance (Duncan's multiple range test). Same alphabets indicate no significant difference (*p*<0.05).
(PDF)

Figure S7 **Chlorophyll retention in tobacco and finger millet plants expressing** ***mtlD*** **gene under methyl viologen coupled with high light-induced oxidative stress.** Leaf segments were taken from transgenic tobacco, finger millet (mtlD-5) and corresponding wild-type plants grown under non-stress condition. These leaf segments were exposed to high light (800 µmol m^−2^ s^−1^) stress with methyl viologen as described in materials and methods section. At the end of stress period, total chlorophyll was measured and percent reduction in total chlorophyll over their corresponding non-stress control was calculated. Values are mean of three replications and the error bar represents standard error. Alphabets above bar indicates the statistical significance (ANNOVA). Same alphabets indicate no significant difference (*p*<0.05).
(PDF)

Figure S8 **Comparison of different features of genetic transformation protocols in finger millet reported in previous literature.** In the literature, three research groups have demonstrated genetic transformation in finger millet. First, particle gun-mediated protocol was developed by Latha and co-workers. Second, *Agrobacterium*-mediated protocol was developed by Antony Ceasar and Ignacimuthu. Both protocol uses shoot apex as explants. Third, Sharma et al., and Jagga-Chugh et al., have demonstrated *UidA* gene expression. In this pictorial representation, different features of these protocols are compared with the protocols developed from this current study.
(PDF)

Table S1 **Standardization of hormonal concentrations for efficient callus induction in finger millet (var. Indaf 9).**
(PDF)

Table S2 **Standardization of hormonal concentrations for efficient shoot induction from the callus in finger millet (var. Indaf 9).**
(PDF)

Table S3 **Efficiency of** ***Agrobacterium*** **-mediated finger millet transformation.**
(PDF)

Table S4 **Comparison of mtlD gene expressing plant species reported in the literature with their corresponding wild-type plants for their abiotic stress tolerance.**
(PDF)
