# Supplementary figures and images for: Stable Expression of mtlD Gene Imparts Multiple Stress Tolerance in Finger Millet
Source: PLoS One. 2014 Jun 12;9(6):e99110. doi: 10.1371/journal.pone.0099110 (PMC4055669; doi:10.1371/journal.pone.0099110)

Figure S1

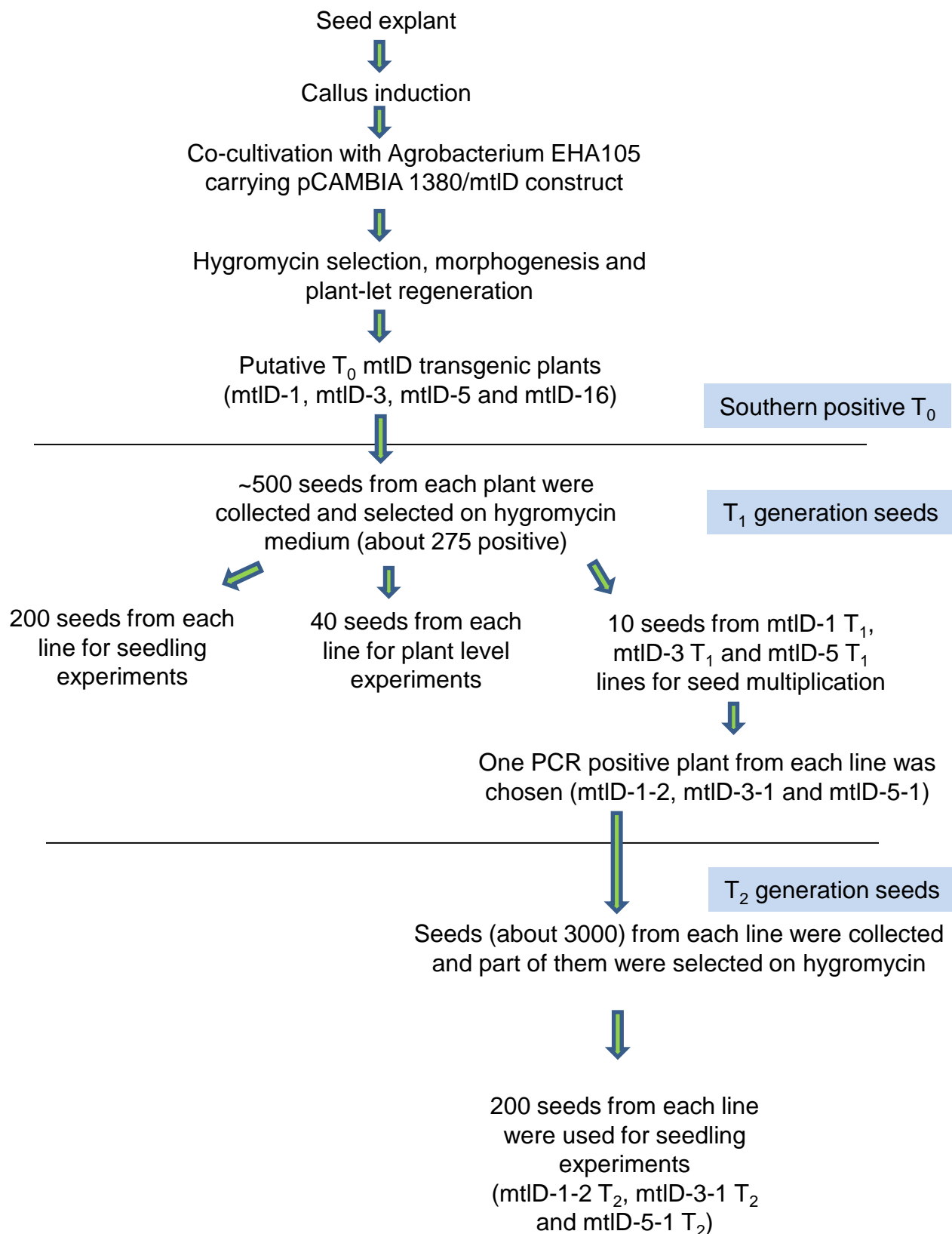

Supplement: Figure S1 — Steps involved in development of finger millet transgenic plants expressing mtlD gene. The T0 transgenic plants (var. GPU28) which were confirmed by PCR and Southern blot were grown in greenhouse and seeds were collected. For further experiments, out of 20 putative transgenic events, four T0 transgenic events were used. These four events were designated as mtlD-1, mtlD-3, mtlD-5, mtlD-16 and taken forward. Seeds obtained from three (T0) events (mtlD-1, mtlD-3 and mtlD-5) were used for further experiments. Seeds from each event were bulked and used for seedling experiment (named as mtlD-1 T1, mtlD-3 T1 and mtlD-5 T1) or plant level experiment. Plants obtained from individual seeds representing each (T1) event were assessed for presence of mtlD gene by PCR and then seeds were collected from these plants. These seeds were designated as mtlD-1-2 T2, mtlD-3-1 T2 and mtlD-5-1 T2 and used for mannitol quantification. A sub-set of seedlings obtained from these seeds were used for stress experiments. (PDF) [file pone.0099110.s001.pdf]

Figure S2

**A**

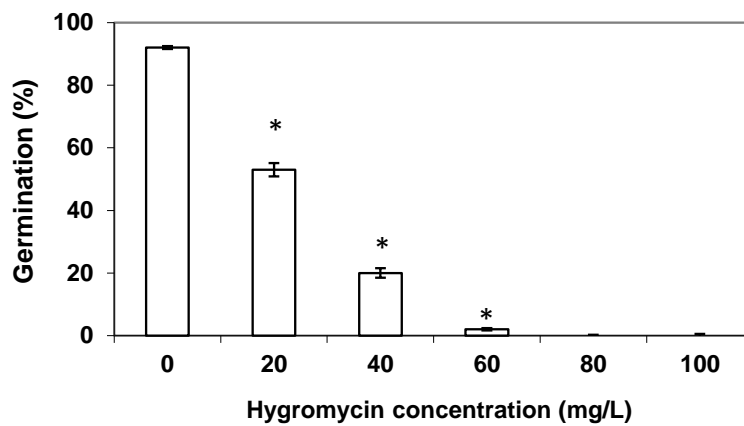

**B**

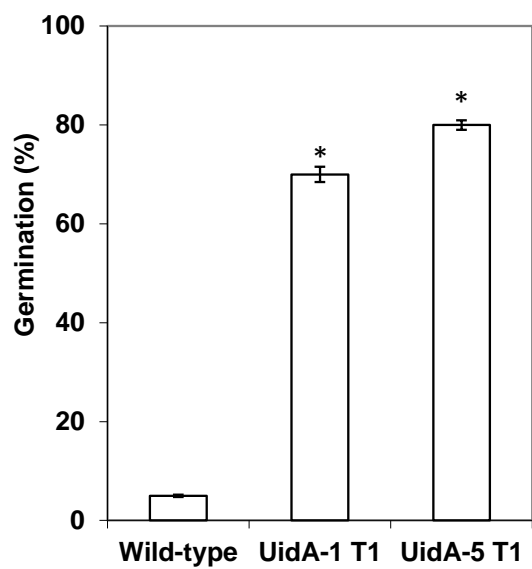

Supplement: Figure S2 — Germination of seeds obtained from UidA gene expressing finger millet transgenic plants on hygromycin. Wild-type (var. GPU28) finger millet seeds were germinated in petri dishes on filter paper with different concentration of hygromycin for 5 days at 30°C with 70% relative humidity in dark and germination percentage was recorded at the end of treatment period (A). Similarly, seeds obtained from the GUS positive transgenic finger millet plants (UidA-1; T1 generation) and wild-type (var. GPU28) were germinated on hygromycin (60 mg/L) and number of seedlings survived was recorded (B). Asterisks indicate values are statistically significant (student's t test; p<0.05) versus corresponding wild-type. Each bar represents the mean of standard error values (n = 10). (PDF) [file pone.0099110.s002.pdf]

Figure S3

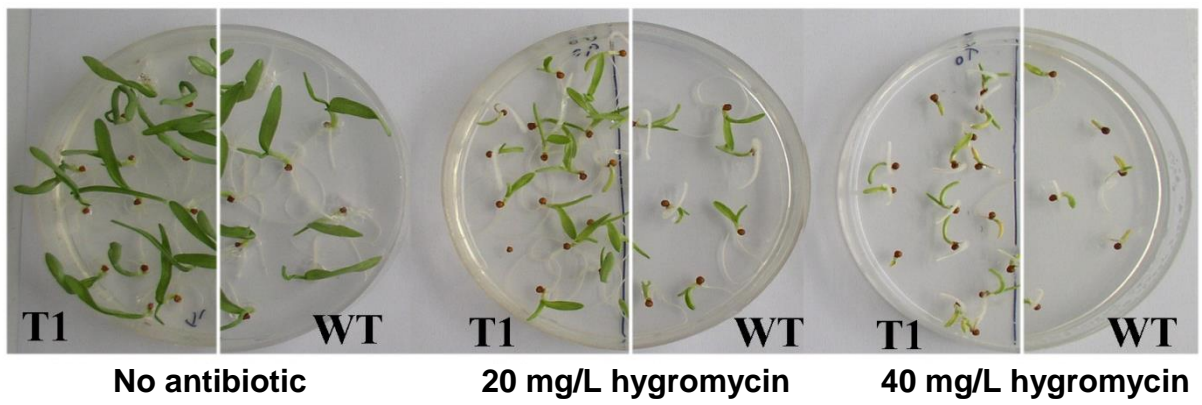

Supplement: Figure S3 — Growth of mtlD expressing finger millet transgenic (T1 generation) and wild-type seedlings on hygromycin medium. Seeds from mtlD transgenic finger millet (T1; mtlD-1 T1 generation) and wild-type (WT, var. GPU28) were inoculated on the MS medium containing hygromycin (20 mg/L or 40 mg/L) for five days. Seedling growth on antibiotic medium was photographed at the end of treatment period. (PDF) [file pone.0099110.s003.pdf]

Figure S4

**A**

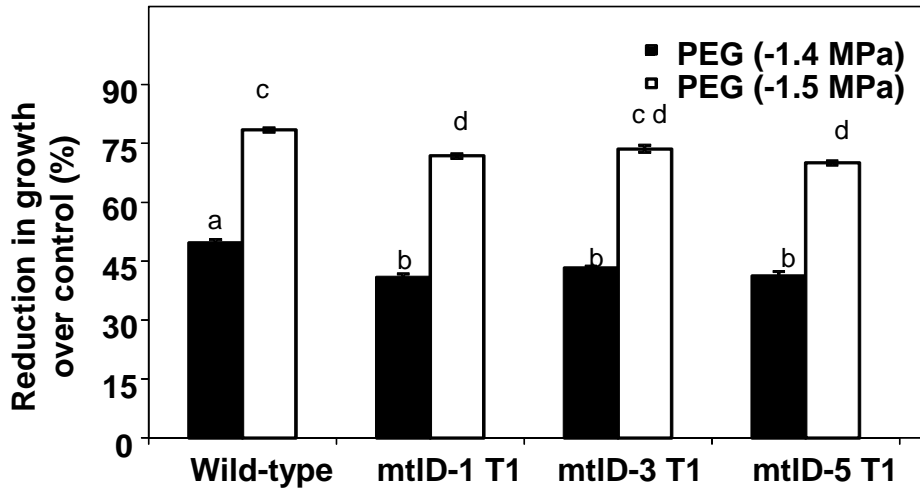

**B**

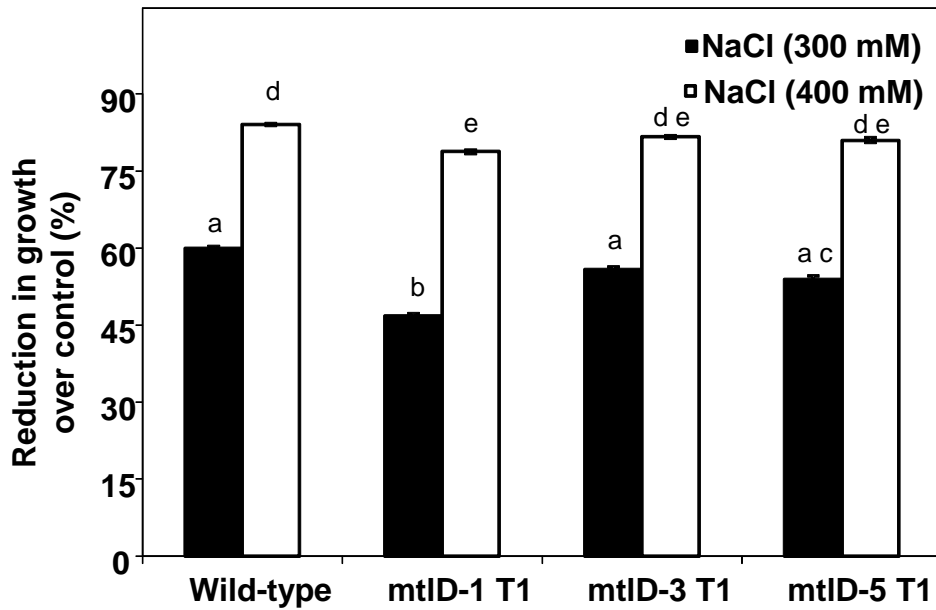

**C**

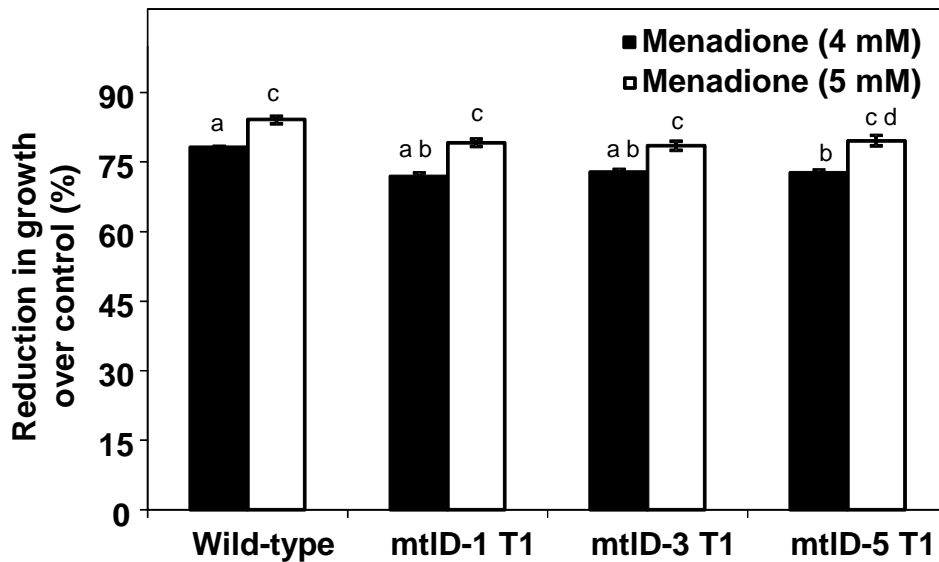

Figure S4 (continued)

D

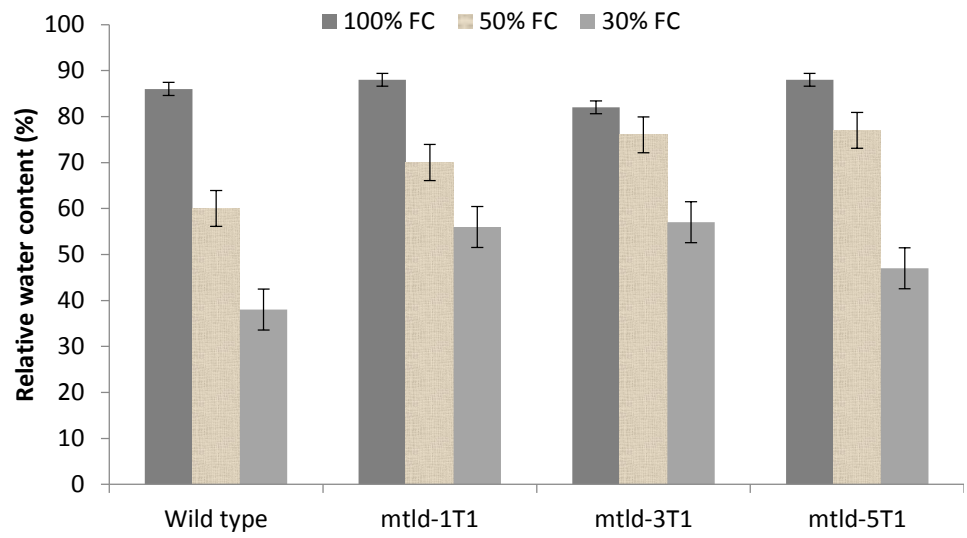

Supplement: Figure S4 — Performance of mtlD expressing finger millet transgenic seedlings under osmotic, salinity and oxidative stress. Finger millet transgenic (T1 generation) and wild-type (var. GPU28) seedlings (1.5 cm length) were initially acclimated with lower concentration of corresponding stresses (−0.2 MPa PEG, 50 mM NaCl, and 1 mM menadione) for 8 h and then subjected to indicated concentrations of respective severe stress levels for 48 h. Seedlings were allowed to recover for two days and recovery growth of osmotic stress (A), salinity stress (B) and menadione induced oxidative stress (C) were measured and percent reduction in growth over corresponding control was calculated. The percent relative water content was measured from the plants exposed to 100%, 50% and 30% field capacity (D). Each bar represents the mean of standard error values (n = 20). Experiments were repeated twice. Alphabets above bar indicates the statistical significance (ANNOVA). Same alphabets indicate no significant difference (p<0.05). (PDF) [file pone.0099110.s004.pdf]

Figure S5

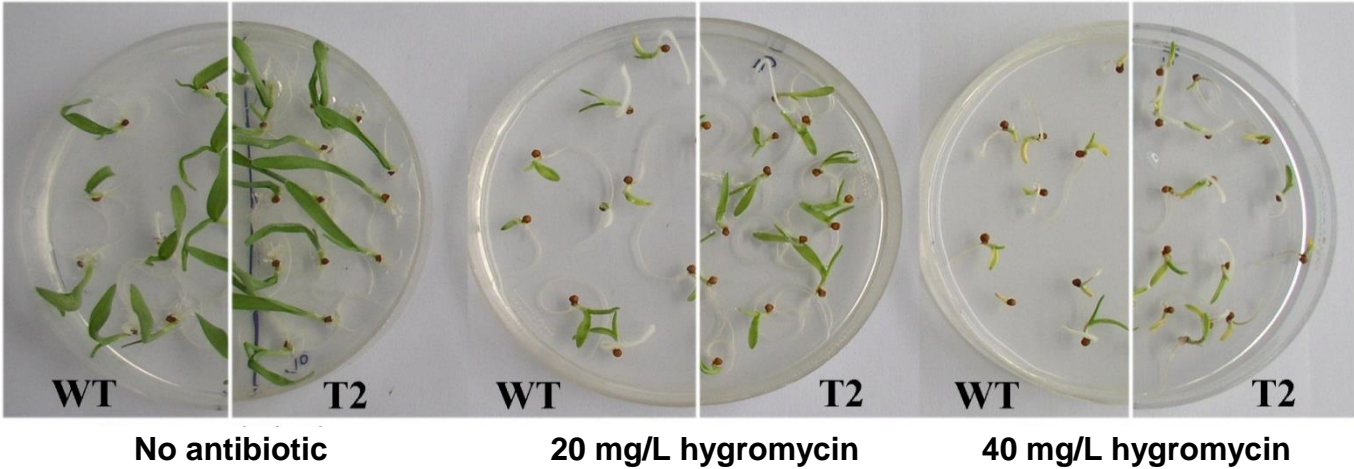

Supplement: Figure S5 — Growth of mtlD expressing finger millet transgenic (T2 generation) and wild-type seedlings on hygromycin medium. Seeds from mtlD expressing transgenic finger millet (mtlD-1-2 T2) and wild-type (WT, var. GPU28) were incubated on the MS medium containing hygromycin (20 mg/L or 40 mg/L) for five days. Seedling growth on antibiotic medium was photographed at the end of treatment period. (PDF) [file pone.0099110.s005.pdf]

Figure S6

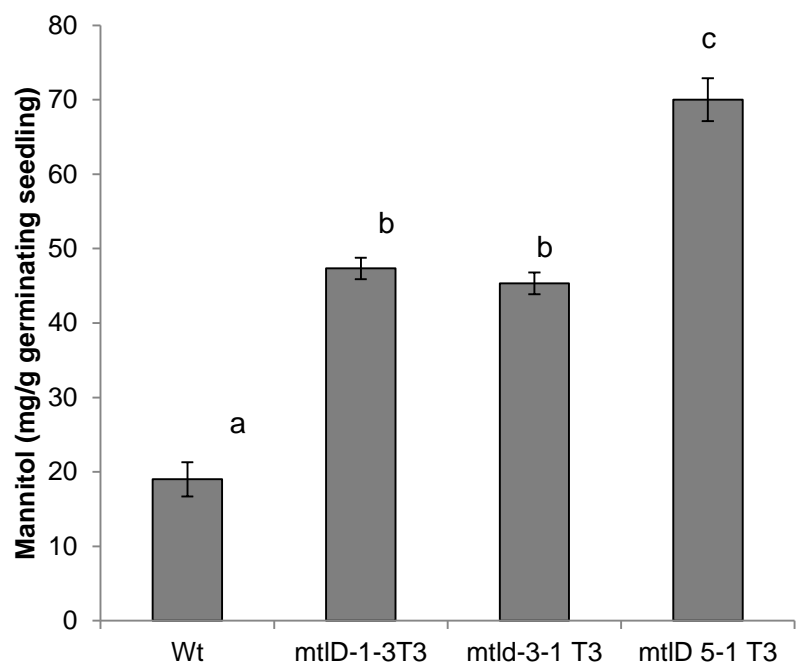

Supplement: Figure S6 — Mannitol accumulation in mtlD gene expressing finger millet germinating seedlings. Mannitol content in the seedlings obtained from T2 generation plants were estimated as described in material and methods. Alphabets above bar indicates the statistical significance (Duncan's multiple range test). Same alphabets indicate no significant difference (p<0.05). (PDF) [file pone.0099110.s006.pdf]

Figure S7

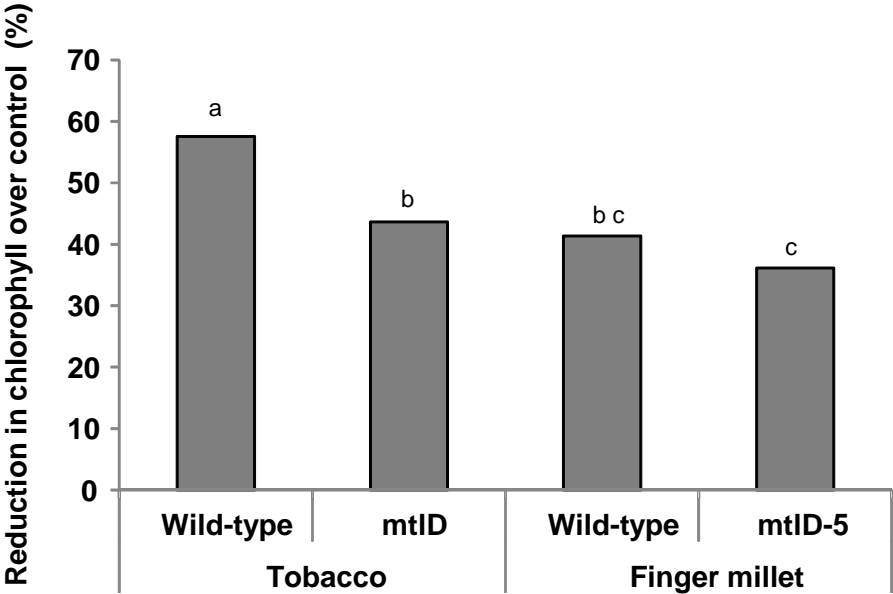

Supplement: Figure S7 — Chlorophyll retention in tobacco and finger millet plants expressing mtlD gene under methyl viologen coupled with high light-induced oxidative stress. Leaf segments were taken from transgenic tobacco, finger millet (mtlD-5) and corresponding wild-type plants grown under non-stress condition. These leaf segments were exposed to high light (800 µmol m−2 s−1) stress with methyl viologen as described in materials and methods section. At the end of stress period, total chlorophyll was measured and percent reduction in total chlorophyll over their corresponding non-stress control was calculated. Values are mean of three replications and the error bar represents standard error. Alphabets above bar indicates the statistical significance (ANNOVA). Same alphabets indicate no significant difference (p<0.05). (PDF) [file pone.0099110.s007.pdf]
