# Supplementary material for: Stable Expression of mtlD Gene Imparts Multiple Stress Tolerance in Finger Millet
Source: PLoS One. 2014 Jun 12;9(6):e99110. doi: 10.1371/journal.pone.0099110 (PMC4055669; doi:10.1371/journal.pone.0099110)
Supplement: Figure S8 — Comparison of different features of genetic transformation protocols in finger millet reported in previous literature. In the literature, three research groups have demonstrated genetic transformation in finger millet. First, particle gun-mediated protocol was developed by Latha and co-workers. Second, Agrobacterium-mediated protocol was developed by Antony Ceasar and Ignacimuthu. Both protocol uses shoot apex as explants. Third, Sharma et al., and Jagga-Chugh et al., have demonstrated UidA gene expression. In this pictorial representation, different features of these protocols are compared with the protocols developed from this current study. (PDF) [file pone.0099110.s008.pdf]

Figure S8

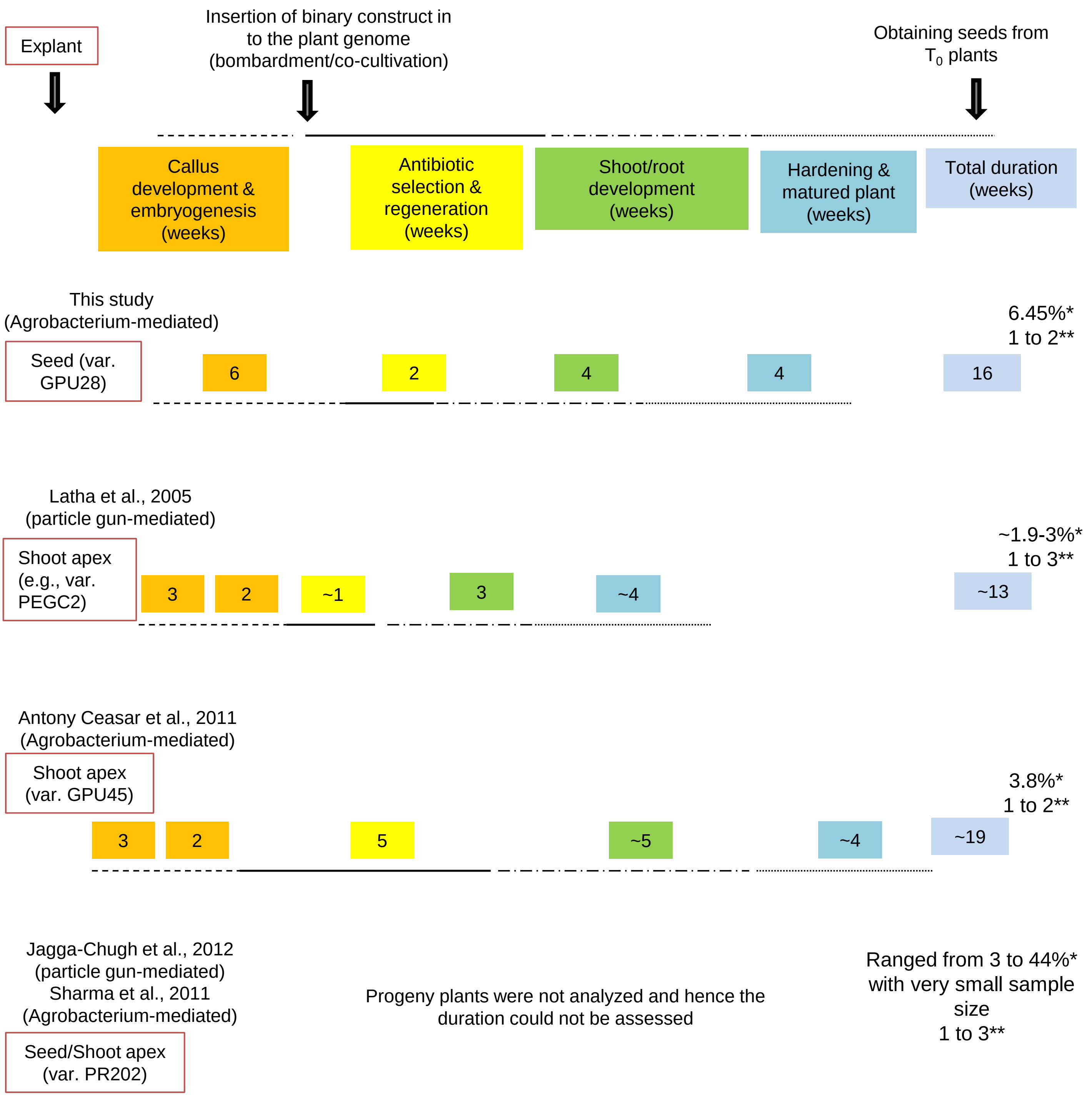

*transformation efficiency
**copy number of transgene integrated into genome
~denotes calculated theoretical values (authors did not mention the actual values) and the sample number was small (<20)
Numbers given in filled boxes indicate duration in weeks

Figure S6 (continued)

References:

1. Antony Ceasar S, Ignacimuthu S (2011) Agrobacterium-mediated transformation of finger millet (*Eleusine coracana* (L.) Gaertn.) using shoot apex explants. Plant Cell Reports 30: 1759-1770.
2. Latha AM, Rao KV, Reddy VD (2005) Production of transgenic plants resistant to leaf blast disease in finger millet (*Eleusine coracana* (L.) Gaertn.). Plant Science 169: 657-667.
3. Jagga-Chugh S, Kachhwaha S, Sharma M, Kothari-Chajer A, Kothari SL. 2012. Optimization of factors influencing microprojectile bombardment-mediated genetic transformation of seed-derived callus and regeneration of transgenic plants in *Eleusine coracana* (L.) Gaertn. *Plant Cell Tissue and Organ Culture* 109(3): 401-410.
4. Sharma M, Kothari-Chajer A, Jagga-Chugh S, Kothari SL. 2011. Factors influencing *Agrobacterium tumefaciens*-mediated genetic transformation of *Eleusine coracana* (L.) Gaertn. *Plant Cell Tissue and Organ Culture* **105**(1): 93-104.
