# Supplementary material for: Stable Expression of mtlD Gene Imparts Multiple Stress Tolerance in Finger Millet
Source: PLoS One. 2014 Jun 12;9(6):e99110. doi: 10.1371/journal.pone.0099110 (PMC4055669; doi:10.1371/journal.pone.0099110)
Supplement: Table S1 — Standardization of hormonal concentrations for efficient callus induction in finger millet (var. Indaf 9). (PDF) [file pone.0099110.s009.pdf]

Supporting information

Table S1. Standardization of hormonal concentrations for efficient callus induction in finger millet (var. Indaf 9).

**Medium composition**	**17***	**19***	**21***	**23***	**25***
Control (MS media without hormones)	0	0	0	0	0
MS + 0.5 mg/L BA + 1.0 mg/L 2,4-D	211	254	289	350	430
MS + 0.5 mg/LBA + 1.5 mg/L2,4-D	207	347	395	460	440
MS + 0.5 mg/LBA + 2.0 mg/L2,4-D	212	351	366	450	440
MS + 0.5 mg/LBA + 2.5 mg/L2,4-D	205	340	369	360	379
MS + 0.5 mg/LBA + 3.0 mg/L2,4-D	245	376	487	500	570
MS + 1.0 mg/LBA + 1.0 mg/L2,4-D	177	268	277	341	460
MS + 1.0 mg/LBA + 1.5 mg/L2,4-D	215	290	333	399	450
MS + 1.0 mg/LBA + 2.0 mg/L2,4-D	200	274	360	360	440
MS + 1.0 mg/LBA + 2.5 mg/L2,4-D	209	266	350	409	380
MS + 1.0 mg/LBA + 3.0 mg/L2,4-D	199	256	360	410	430

*days after inoculation of seeds

**all values in the table represent callus weight (mg)/ seed callus
