# Supplementary material for: Stable Expression of mtlD Gene Imparts Multiple Stress Tolerance in Finger Millet
Source: PLoS One. 2014 Jun 12;9(6):e99110. doi: 10.1371/journal.pone.0099110 (PMC4055669; doi:10.1371/journal.pone.0099110)
Supplement: Table S2 — Standardization of hormonal concentrations for efficient shoot induction from the callus in finger millet (var. Indaf 9). (PDF) [file pone.0099110.s010.pdf]

Table S2. Standardization of hormonal concentrations for efficient shoot induction from the callus in finger millet (var. Indaf 9).

**Treatment**	**Average shoot number per similar size callus**	**Average shoot length of shoot-lets from the previous column (cm)**
Control (MS media without hormones)	6	2.75
MS + 0.5 mg/L BA	16	3.2
MS + 1.0 mg/L BA	23	4.3
MS + 0.5 mg/L NAA+ 0.5 mg/L BA	10	6.8
MS + 1.0 mg L-1 NAA+ 0.5 mg/L BA	9	8.3
