# Supplementary material for: Stable Expression of mtlD Gene Imparts Multiple Stress Tolerance in Finger Millet
Source: PLoS One. 2014 Jun 12;9(6):e99110. doi: 10.1371/journal.pone.0099110 (PMC4055669; doi:10.1371/journal.pone.0099110)
Supplement: Table S3 — Efficiency of Agrobacterium -mediated finger millet transformation. (PDF) [file pone.0099110.s011.pdf]

Table S3. Efficiency of Agrobacterium-mediated finger millet transformation

Sl. No.	Experiment	Number of calli infected	Number of calli survived on antibiotic medium	Number of calli regenerated into shoots	Transformation efficiency (%)
1	*UidA* gene transformation*	73	7	4	5.4**
2	*mtlD* gene transformation*	80	9	6	7.5**
**Total**		153	16	10	6.45
**SD**			1.41	1.48	1.48

*finger millet variety GPU28 was used in these studies
**values are given for one set of experiment. Similar results were found in second independent experiment.
