# Supplementary material for: Stable Expression of mtlD Gene Imparts Multiple Stress Tolerance in Finger Millet
Source: PLoS One. 2014 Jun 12;9(6):e99110. doi: 10.1371/journal.pone.0099110 (PMC4055669; doi:10.1371/journal.pone.0099110)
Supplement: Table S4 — Comparison of mtlD gene expressing plant species reported in the literature with their corresponding wild-type plants for their abiotic stress tolerance. (PDF) [file pone.0099110.s012.pdf]

**Table S4. Comparison of *mtlD* gene expressing plant species reported in the literature with their corresponding wild-type plants for their abiotic stress tolerance.**

| | Increase in tolerance level (%) over wild-type* | | | | | |
|---|---|---|---|---|---|---|
| **Plant species** | **Parameter used for assessing tolerance level**** | **Drought stress** | **Salinity stress** | **Oxidative stress** | **Treatment details, possible mechanism and other comments** | **Reference** |
| C3 plant species | | | | | | |
| Tobacco | Stem length<br>$CO_2$ assimilation<br>Membrane leakage | 54 | - | <br><br>2.8<br>37.5 | • Increased osmotic adjustment played a role in drought tolerance<br>• High light induced oxidative stress<br>• Methylviologen-mediated oxidative stress<br>• Increase in chilling tolerance was 10.4 | [1] |
| Tobacco | $CO_2$ assimilation | 33 | - | - | Oxidative stress by methylviologen coupled with drought stress also showed similar results | [2] |
| Tobacco | Plant growth (biomass) | - | 53.3 | - | One of the earliest reports demonstrating possibility of increasing abiotic stress tolerance in plants by expressing *mtlD* gene | [3] |

| | | | | | | |
|---|---|---|---|---|---|---|
| Tobacco (var. KST19) | Total chlorophyll reduction | - | - | 13.9 | Plant level experiment | [4] (this study) |
| Egg plant | Seedling weight | 66.6-82.3 | 50-88.8 | - | 10% PEG was used<br>200 mMNaCl was used | [5] |
| Arabidopsis | Seed germination | - | 45 | - | 200 mMNaCl was used | [6] |
| Tomato | Biomass<br>Seed germination | 28.5 | 42 | - | 10% PEG was used<br>50 mMNaCl was used | [7] |
| Potato | | - | 59.2 | - | • 100 mMNaCl was used<br>• Cellular protection (via free radical removal or macro molecules protection) might be reason for increased tolerance of transgenic seedlings | [8] |
| Petunia | Root/shoot dry weight | - | - | - | Increase in chilling stress tolerance was 5.19-16.33; Increased osmotic adjustment played a role | [9] |
| Wheat | Shoot dry weight | 54.1 | 50 | - | • Osmotic adjustment did not play role in drought tolerance<br>• Protection from free radical might be reason for increased tolerance | [10] |

| | | | | | | |
|---|---|---|---|---|---|---|
| Rice | Shoot height<br>Biomass | - | 77-141<br>17-50 | - | - | [11] |
| C4 plant species | | | | | | |
| Sorghum | Leaf water content<br>Biomass | 2.6 | <br><br>17-23 | - | • PEG assay<br>• 200 mMNaCl stress | [12] |
| Finger millet | Seedling growth | 8-10 | 4-7 | 5-6 | Seedling experiment [mtlD-5-1 T2] | [4] (this study) |

*calculated value
**also indicate the experimental data in the manuscript considered for calculations of increased tolerance

## References


1. Macaluso L, Lo Bianco R, Rieger M (2007) Mannitol-producing tobacco exposed to varying levels of water, light, temperature and paraquat. Journal of Horticultural Science & Biotechnology 82: 979-985.
2. Alam B, Jacob J, Earl HJ (2010) Photosynthetic efficiency of transgenic tobacco plants (*Nicotiana tabacum* l.) over-expressing *mtld* gene under drought and paraquat stress. Indian Journal of Plant Physiology 15: 186-191.
3. Tarczynski MC, Jensen RG, Bohnert HJ (1993) Stress protection of transgenic tobacco by production of the osmolyte mannitol. Science 259: 508-510.
4. Hema R (2006) Relevance of mannitol in dehydration stress tolerance: development and analysis of the transgenics expressing mtlD in model systems and finger millet (*Eleusine coracana* (l.) gaertn) [PhD]. Bangalore: University of Agricultural Sciences. 197 p.
5. Prabhavathi V, Yadav JS, Kumar PA, Rajam MV (2002) Abiotic stress tolerance in transgenic eggplant (*Solanum melongena* L.) by introduction of bacterial mannitol phosphodehydrogenase gene. Molecular Breeding 9: 137-147.
6. Thomas JC, Sepahi M, Arendall B, Bohnert HJ (1995) Enhancement of seed germination in high salinity by engineering mannitol expression in *Arabidopsis thaliana*. Plant, Cell & Environment 18: 801-806.
7. Khare N, Goyary D, Singh NK, Shah P, Rathore M, et al. (2010) Transgenic tomato cv. Pusa Uphar expressing a bacterial mannitol-1-phosphate dehydrogenase gene confers abiotic stress tolerance. Plant Cell Tissue and Organ Culture 103: 267-277.

8. Rahnama H, Vakilian H, Fahimi H, Ghareyazie B (2011) Enhanced salt stress tolerance in transgenic potato plants (*Solanum tuberosum* L.) expressing a bacterial *mtlD* gene. Acta Physiologiae Plantarum 33: 1521-1532.
9. Chiang YJ, Stushnoff C, McSay AE (2005) Overexpression of mannitol-1-phosphate dehydrogenase increases mannitol accumulation and adds protection against chilling injury in petunia. Journal of the American Society for Horticultural Science 130: 605-610.
10. Abebe T, Guenzi AC, Martin B, Cushman JC (2003) Tolerance of mannitol accumulating transgenic wheat to water stress and salinity. Plant Physiology 131: 1748-1755.
11. Su J, Chen P, Wu R (1999) Transgene expression of mannitol-1-phosphate dehydrogenase enhanced the salt-stress tolerance of the transgenic rice seedlings. Scientia Agricultura Sinica 32: 101-103.
12. Maheswari M, Varalaxmi Y, Vijayalakshmi A, Yadav SK, Sharmila P, et al. (2010) Metabolic engineering using *mtlD* gene enhances tolerance to water deficit and salinity in sorghum. Biologia Plantarum 54: 647-652.
